# Supplementary material for: The association between nurse staffing levels and the timeliness of vital signs monitoring: a retrospective observational study in the UK
Source: BMJ Open. 2019 Sep 27;9(9):e032157. doi: 10.1136/bmjopen-2019-032157 (PMC6773325; doi:10.1136/bmjopen-2019-032157)
Supplement: Supplementary data [file bmjopen-2019-032157supp001.pdf]

# SUPPLEMENTARY MATERIAL

## A1 Summary of study hospital escalation and vital sign monitoring protocol

| Early warning Score | Risk category | Max interval between observations | Nurse Actions | Doctor Actions |
|---|---|---|---|---|
| **0-1** | Low | 6 hours /<br>12 Hours If stable for 6 hours | (None specified – observations as per schedule) | |
| **2** | Low | 6 hours | (None specified – observations as per schedule) | |
| **3-5** | Medium | 4 hours | Inform nurse in charge | |
| **<6, but with one or more individual triggers** | High | 4 hours | Registered nurse to inform doctor (FY2 / SHO) | See patient within 2 hours |
| **6** | High | 4 hours | Registered nurse to inform doctor (FY2 / SHO) | See patient within 2 hours |
| **7-8** | High | 1 hour | Registered nurse to inform doctor (FY2 / SHO)<br>Consider continuous monitoring | See patient within 30 minutes<br>Call SpR / outreach (after 8.30 SpR / ICU) |
| **9+** | Critical | 30 minutes | Registered nurse to inform doctor (SpR)<br>Consider continuous monitoring | See patient within 15 minutes<br>Call SpR / outreach (after 8.30 SpR / ICU) |

*Extreme values on any one parameter may trigger a higher level of escalation than otherwise indicated*
*Full policy can be found at :*
*http://www.porthosp.nhs.uk/about-us/policies-and-guidelines/policies/Clinical/Deteriorating%20Patient%20Policy%20-%20Management.doc (accessed 13/1/2018).*
*Adapted from Griffiths P, Ball J, Bloor K, et al. Nurse staffing levels, missed vital signs and mortality in hospitals: retrospective longitudinal observational study. Heal Serv Deliv Res 2018;6:1–120.*

**A2 Study flowchart**

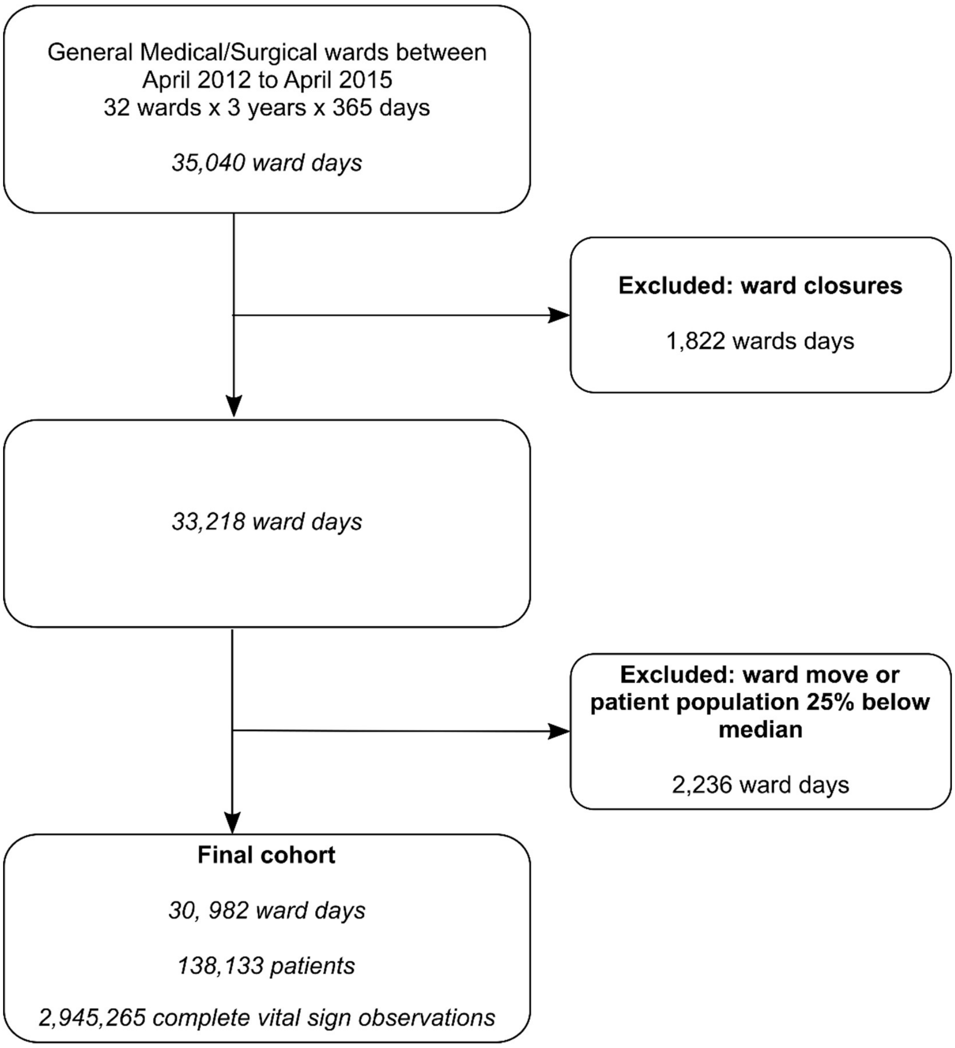

### A3 Mean staffing per ward

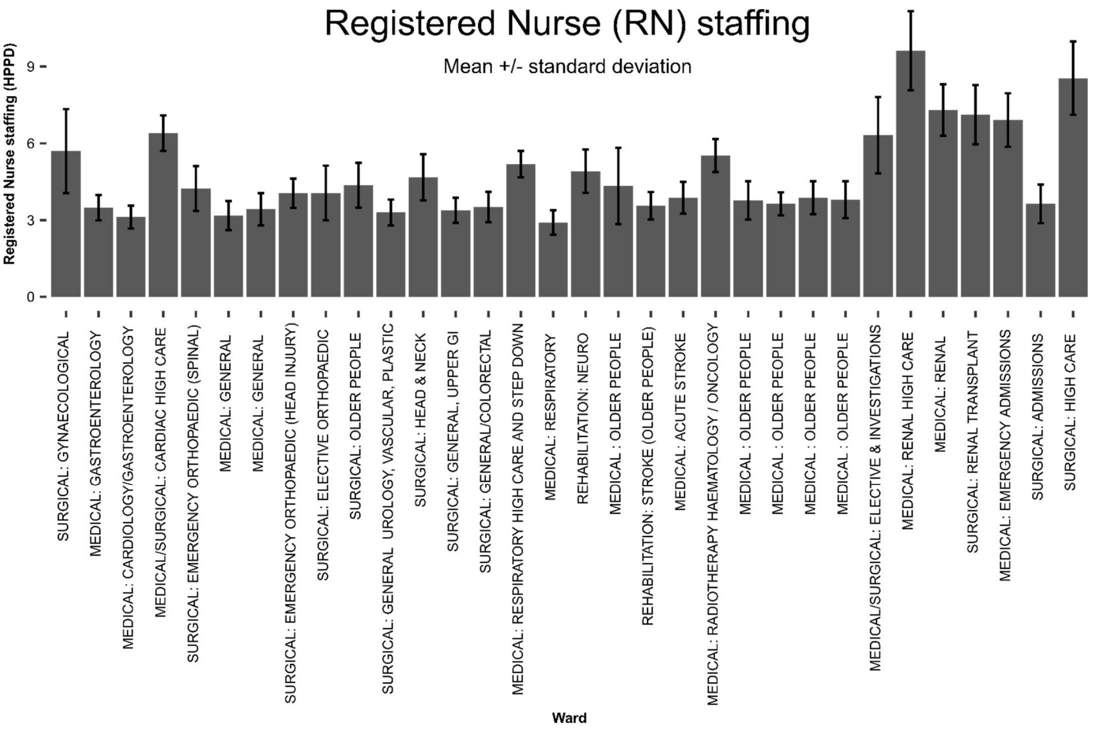


### A4 Missed observations (low and medium acuity)

The tables below show the relationship of staffing levels with respect to the primary outcome (missed observations) for low and medium acuity observations.

*Table A4a Low acuity*

| | IRR | 95% confidence Interval | p-value |
|---|---|---|---|
| **RN staffing** | 0.98 | 0.973-0.986 | < 0.001 |
| **NA staffing** | 0.933 | 0.926-0.939 | < 0.001 |
| **Patient turnover** | 1.04 | 1.03-1.04 | < 0.001 |
| **Higher acuity patients** | 2.03 | 1.93-2.14 | < 0.001 |
| **RN staffing x NA staffing** | 1.02 | 1.02-1.02 | < 0.001 |

*Table A4b Medium acuity*

| | IRR | 95% confidence Interval | p-value |
|---|---|---|---|
| **RN staffing** | 0.977 | 0.971-0.984 | < 0.001 |
| **NA staffing** | 0.964 | 0.957-0.971 | < 0.001 |
| **Patient turnover** | 0.989 | 0.984-0.995 | < 0.001 |
| **Higher acuity patients** | 0.641 | 0.609-0.676 | < 0.001 |
| **RN staffing x NA staffing** | 1.01 | 1.00-1.01 | < 0.001 |

## A5 Delayed observations

The tables below show the relationship of staffing levels with respect to the secondary outcome (delayed observations).

*Table A5a All observations*

| | IRR | 95% confidence Interval | p-value |
|---|---|---|---|
| **RN staffing** | 0.984 | 0.981-0.987 | < 0.001 |
| **NA staffing** | 0.98 | 0.976-0.983 | < 0.001 |
| **Patient turnover** | 1 | 1.00-1.01 | 0.0186 |
| **Higher acuity patients** | 2.23 | 2.18-2.28 | 0 |
| **RN staffing x NA staffing** | 1.01 | 1.00-1.01 | < 0.001 |

*Table A5b High acuity observations*

| | IRR | 95% confidence Interval | p-value |
|---|---|---|---|
| **RN staffing** | 0.987 | 0.978-0.996 | 0.0043 |
| **NA staffing** | 1 | 0.993-1.01 | 0.55 |
| **Patient turnover** | 0.996 | 0.987-1.00 | 0.38 |
| **Higher acuity patients** | 1.03 | 0.960-1.10 | 0.415 |
| **RN staffing x NA staffing** | 1 | 0.995-1.00 | 0.943 |

## A6 Missed observations (tertiles of nurse staffing levels)

The table below shows the model used to explore interactions between NA and RN staffing groups (see Figure 1 in main manuscript).

*Table A6 Mixed-effects Poisson regression with staffing variables modelled as tertiles: Association between staffing and all missed observations AIC: 215,999 BIC: 216,099*

| | IRR | 95% confidence interval | p-value |
|---|---|---|---|
| **RN staffing Q2** | 0.974 | 0.963-0.985 | p < 0.001 |
| **RN staffing Q3** | 0.934 | 0.923-0.946 | p < 0.001 |
| **NA staffing Q2** | 0.92 | 0.910-0.931 | p < 0.001 |
| **NA staffing Q3** | 0.919 | 0.908-0.931 | p < 0.001 |
| **Patient turnover** | 1.01 | 1.01-1.02 | p < 0.001 |
| **Higher acuity patients** | 4.83 | 4.68-4.99 | 0 |
| **RN staffing Q2 x NA staffing Q2** | 1.02 | 0.999-1.03 | 0.0603 |
| **RN staffing Q3 x NA staffing Q2** | 1.04 | 1.02-1.06 | p < 0.001 |
| **RN staffing Q2 x NA staffing Q3** | 1.02 | 1.00-1.04 | 0.0525 |
| **RN staffing Q3 x NA staffing Q3** | 1.05 | 1.03-1.07 | p < 0.001 |
