# Supplementary material for: The association between nurse staffing levels and the timeliness of vital signs monitoring: a retrospective observational study in the UK
Source: BMJ Open. 2019 Sep 27;9(9):e032157. doi: 10.1136/bmjopen-2019-032157 (PMC6773325; doi:10.1136/bmjopen-2019-032157)
Supplement: Author's manuscript [file bmjopen-2019-032157.draft_revisions.pdf]

BMJ Open is committed to open peer review. As part of this commitment we make the peer review history of every article we publish publicly available.

When an article is published we post the peer reviewers' comments and the authors' responses online. We also post the versions of the paper that were used during peer review. These are the versions that the peer review comments apply to.

The versions of the paper that follow are the versions that were submitted during the peer review process. They are not the versions of record or the final published versions. They should not be cited or distributed as the published version of this manuscript.

BMJ Open is an open access journal and the full, final, typeset and author-corrected version of record of the manuscript is available on our site with no access controls, subscription charges or pay-per-view fees (http://bmjopen.bmj.com).

If you have any questions on BMJ Open's open peer review process please email info.bmjopen@bmj.com

BMJ Open

The association between nurse staffing levels and the timeliness of vital signs monitoring: a retrospective observational study

Journal:	*BMJ Open*
Manuscript ID	bmjopen-2019-032157
Article Type:	Research
Date Submitted by the Author:	07-Jun-2019

Complete List of Authors:	Redfern, Oliver; Oxford University, Nuffield Department of Clinical Neurosciences<br>Griffiths, Peter; University of Southampton<br>Maruotti, Antonello; Libera Universita Maria Santissima Assunta, Dipartimento di Scienze Economiche<br>Recio-Saucedo, Alejandra; University of Southampton<br>Smith, Gary; University of Bournemouth, School of Health and Social Care

Keywords:	nursing staff, vital signs, patient deterioration

SCHOLARONE™
Manuscripts

The association between nurse staffing levels and the timeliness of vital signs monitoring: a retrospective observational study

AUTHORS

*Oliver Redfern[e,i]

*†Peter Griffiths[a,b,c]

Antonello Maruotti[a, j]

Alejandra Recio-Saucedo[a,b]

Gary Smith[h]

The Missed Care Study Group [+]

[+] The Missed Care Study Group comprises Peter Griffiths (a, b, c, g), Jane Ball (a, b, c), Karen Bloor (d), Dankmar Böhning (a), Jim Briggs (e, g), Chiara Dall'Ora (a, b), Anya De Iongh (f), Jeremy Jones (a), Caroline Kovacs (e), Antonello Maruotti (a), Paul Meredith (b, g), Alejandra Recio-Saucedo (a, b), David Prytherch (e, g), Oliver Redfern (e, i), Paul Schmidt (b, g), Nicky Sinden (g), and Gary Smith (h).

a. University of Southampton, Health Sciences
b. National Institute for Health Research Collaboration for Applied Health Research and Care (Wessex)
c. Karolinska Instutet, Department of Learning, Informatics, Management and Ethics
d. University of York, Health Sciences
e. University of Portsmouth, Centre for Healthcare Modelling and Informatics
f. Independent lay researcher
g. Portsmouth Hospitals NHS Trust, Clinical Outcomes Research Group
h. Bournemouth University, Faculty of Health and Social Sciences
i. Nuffield Department of Clinical Neurosciences, University of Oxford

For peer review only - http://bmjopen.bmj.com/site/about/guidelines.xhtml

j. LUMSA Universita Maria SS. Assunta di Roma, Department of economic science, politics and modern languages

* To be considered as joint first authors

† **Corresponding author**

Professor Peter Griffiths

Chair of Health Services Research

University of Southampton

Room 3021, Building 67, Highfield Campus, Southampton SO17 1BJ

Tel. +44 2380 597877

Email: peter.griffiths@soton.ac.uk

KEYWORDS

Nursing Staff, Hospital/ supply & distribution, Vital signs, Retrospective study, Observational study, patient deterioration

For peer review only - http://bmjopen.bmj.com/site/about/guidelines.xhtml

ABSTRACT

Objectives

Omissions and delays in delivering nursing care are widely-reported consequences of staffing shortages, with potentially serious consequences. However, studies so far have relied exclusively on nurse self-reporting. Monitoring vital signs is a key part of nursing work and electronic recording provides an opportunity to objectively measure delays in care. This study aimed to determine the association between registered nurse (RN) and nursing assistant (NA) staffing levels and adherence to a vital signs monitoring protocol.

Design

Retrospective observational study.

Setting

32 medical and surgical wards in an acute general hospital in England.

Participants

538,238 nursing shifts taken over 30,982 ward days.

Primary and secondary outcome measures

Vital sign observations were scheduled according to a protocol based on the National Early Warning Score (NEWS). The primary outcome was the daily rate of missed vital signs (overdue by ≥67% of the expected time to next observation). The secondary outcome was the daily rate of late vital signs observations (overdue by ≥33%). We undertook sub-group analysis by stratifying observations into low, medium and high acuity using the NEWS score.

Results

Late and missed observations were frequent, particularly in high acuity patients (median = 44%). Higher levels of registered nurse staffing, measured in hours per patient per day (HPPD), were associated with a lower rate of missed observations in all (Incident Rate ratio [IRR] 0.980, 95% Confidence Interval [CI] 0.973-0.986) and high acuity patients (0.982, 0.972-0.992). However, levels of nursing assistant staffing were only associated with the daily rate (0.933, CI 0.926-0.939) of all missed observations.

Conclusions

Adherence to vital sign monitoring protocols is sensitive to levels of nurse and nursing assistant staffing, although high acuity observations appeared unaffected by levels of nursing assistants. We demonstrate that objectively measured omissions in care are related to nurse staffing levels, although the absolute effects are small.

For peer review only - http://bmjopen.bmj.com/site/about/guidelines.xhtml

STUDY REGISTRATION

The data and analyses presented here were part of the larger Missed Care study (ISRCTN registration: 17930973).

STRENGTHS AND LIMITATIONS

- This was a large retrospective cohort study exploring the association between levels of nursing staff in an acute hospital and adherence to a vital signs monitoring protocol.
- In contrast to previous studies that relied on nursing staff self-reporting missed care, we used an objective measure derived from electronically recorded vital signs.
- This study was limited to a single hospital and we were only able to partially adjust for other factors that might affect protocol adherence.

For peer review only

INTRODUCTION

Reports from around the world have highlighted poor nursing care as a cause of avoidable harm [1–3]. Perhaps unsurprisingly, there is mounting evidence that quality of care deteriorates when wards are understaffed [4], yet the extent to which low staffing leads directly to worse outcomes for patients remains in dispute [5]. A number of studies have explored whether nursing work that is delayed or left incomplete (often referred to as “missed care” [6]) provides a plausible causal mechanism leading to worse patient outcomes, as nurses do not have capacity to deliver all required care when staffing levels are inadequate [4,7]. However, details of nursing activities are not always routinely collected or recorded in standard formats, or in systems that can be easily interrogated, by health care providers. Therefore, it is difficult to measure the timing of care or the extent to which care is delivered [8]. Consequently, the evidence supporting an association between missed care and staffing levels is largely based on nurses’ self-reports [4,9,10].

Recording patients’ vital signs is a fundamental aspect of nursing work, and a key component of patient surveillance: infrequent monitoring can cause signs of clinical deterioration to be missed, leading to delays in administering remedial treatment [3,11,12]. A Europe-wide cross-sectional study (RN4CAST) found that 27% of nursing staff reported missing at least some necessary patient surveillance on their last shift [9]. The failure to properly observe and record vital signs observations has been noted as a factor in inquiries into the cause of preventable death in hospital patients [13].

In response to the increasing recognition that monitoring vital signs is sub-optimal, a number of protocols which define observation schedules have been developed and implemented. For example, on general medical and surgical wards, UK guidelines recommend that the frequency of monitoring is directed by the National Early Warning Score (NEWS) [12]. This is a score that provides a composite measure of patients’ physiological abnormalities, based on vital signs measurements: in general, the higher the score, the more frequently patients should be observed. Internationally, a range of similar early warning or escalation systems are used to guide the observation and escalation of care for at-risk patients [14]. However, retrospective studies have shown, at best, partial adherence to monitoring protocols, particularly at night [15,16].

Inadequate staffing is one possible explanation for this lack of adherence, as it may reduce nurses’ capacity to monitor and intervene to prevent deterioration. This could be one explanation for the association between low nurse staffing levels and increased mortality, which has been demonstrated in many studies worldwide [5]. Yet, existing studies linking low staffing to missed care have exclusively used self-report by nurses derived from cross

For peer review only - http://bmjopen.bmj.com/site/about/guidelines.xhtml

sectional surveys [4]. Such studies suffer a number of limitations, including common-method bias, because all variables are derived from the same self-report survey [17].

In this retrospective observational study of an acute hospital in England, we used routinely collected records of vital signs and other clinical and administrative data, including the electronic rostering database, to investigate whether adherence to the hospital's vital signs monitoring protocol was sensitive to the daily levels of nursing staff.

METHODS

Study Design and Setting

This was a retrospective longitudinal observational study of 32 wards in a large acute hospital in the South of England over three years (April 2012 to March 2015).

Data sources and linkage

Sources

This study combined data from four sources. Information on patients (admissions, ward transfers) was obtained from the Patient Administration System (PAS), allowing us to calculate bed occupancy and the number of admissions to each ward. Vital signs observations were obtained from the Vitalpac™ system [18]. Data items were: anonymised admission identifier, time of observation, National Early Warning Score (NEWS) [12], time to next observation.

Levels of nurse staffing were derived from two source databases. For standard contractual shifts, we extracted data from an electronic rostering system, detailing the date, location, number of hours and grade of each nurse for every shift. The second source was a similar database recording all bank (extra contractual work by staff employed by the hospital) and agency (staff employed through an external agency) shifts.

In total we identified 538,238 shifts worked over the study period by either registered nurses (RNs; fully qualified nurses on the Nursing and Midwifery Council Register with university diploma or degree level qualification or equivalent) or NAs (nursing assistant personnel with no formal training requirements or registration, typically employed in roles described as health care assistants in NHS pay bands 2-4). We did not have access to data on shifts undertaken by student nurses. However, they are considered supernumerary for the purposes for staff allocation.

Linkage

Nursing shifts worked on each day of the study were linked to vital signs observations and admission data (from PAS) using ward location identifiers and time stamps. For each of the

For peer review only - http://bmjopen.bmj.com/site/about/guidelines.xhtml

For peer review only

32 wards, we calculated daily patient and staffing levels. From a theoretical maximum of 35,040 ward days (365 days x 3 years x 32 wards) there were 1,822 ward days where one or more of the study wards was closed and 2,236 (6.4%) wards where we could not properly match patient records and staffing. This was generally when wards relocated and nursing shifts were still recorded in the old location for a while. Additionally, periods when wards opened, closed or transferred were often associated with unusual values for patient numbers or staff/patient ratios due to low patient census or delayed recording of staff transfers to the new unit. Therefore, we excluded all ward days where the patient census fell below 25% of the ward median. We were unable to link e-roster to the staff taking the observations as no standard identifier was available although we attempted to identify the grade of staff taking the observations using a descriptive field in the Vitalpac™ system.

Outcomes

A total of 2,864,975 complete sets of vital signs were available for analysis. The primary outcome of the study was *missed* vital signs observations. The secondary outcome was *delayed* observations.

Both outcomes were calculated with reference to the hospital's vital signs monitoring protocol. The protocol is based on the National Early Warning Score (NEWS)[12], where the level of derangement in vital signs (the NEWS value) is aggregated into a single integer. This is then used to determine when the patient should next be observed – in general, higher scores prompt more frequent observation. For example, if the NEWS value is 2, the patient should be observed at least every 6 hours. Patients with the lowest score (NEWS = 0) are observed every 12 hours and those with higher scores more frequently.

We defined a vital signs observation as *missed* if overdue by more than 67% of the expected time to next observation determined by the previous NEWS value. Similarly, an observation was *delayed* if overdue by more than 33% of the expected time to next observation determined by the previous NEWS value. For example, if the next observation was due in 60 minutes, it was classified as *delayed* if taken > 80 minutes after the previous observation and *missed* if taken > 100 minutes later.

For sub-group analyses, missed/delayed observations were further stratified in acuity categories according to the previous NEWS value as follows:

- Low: where previous NEWS value < 3
- Medium: where previous NEWS value was between 3-5
- High: where previous NEWS value > 5

For peer review only - http://bmjopen.bmj.com/site/about/guidelines.xhtml

Exposures

For each study day on each ward, we calculated the average staffing levels in Hours per Patient Day (HPPD) for both RNs and NAs. HPPD was calculated by dividing the total number of nursing hours worked by the daily bed occupancy (for that ward). Daily bed occupancy was calculated from the PAS database where a value of one indicates a single bed being occupied continuously for one day. A HPPD of 24 indicates one-to-one nursing.

To account for variations in other aspects of nursing workload, we derived variables to quantify admission rates ("patient turnover") and the proportion of observations that were for patients requiring 4-hourly or more frequent observation on each day of the study (i.e. patient with NEWS value ≥3, "higher acuity"). Patient turnover was calculated by dividing total daily RN staffing (in days) by the number of new admissions.

Statistical methods

We chose mixed-effects Poisson regression as our modelling framework to examine the relationship between missed/delayed observations and staffing. Random effects terms were introduced for each ward. All other co-variates were added as fixed effects in the models. Where not otherwise stated, all summary measures are reported using median and interquartile range. All analyses were undertaken using the R statistical environment v3.5 [19] and mixed-effects models were fit using the lme4 package [20].

By modelling the effect of each staff group separately, we considered the extent to which the labour inputs from one group might substitute for the other. Additionally, we tested for potential that NAs acted as labour complements, enhancing the effectiveness of RNs by adding interaction terms to each model. We assessed whether these terms improved model fit by examining the Akaike information criterion (AIC) and Bayesian information criterion (BIC).

Ethical approval

The study was approved by the National Research Ethics Service. East Midlands – Northampton Committee Ref: 15/EM/0099. All identifiable information for patients and staff was removed at source. Internal identifiers were anonymised prior to transfer to the research database. Consequently, it was not possible for the research team to identify participants in the study.

Patient and Public Involvement

As part of the parent study, we undertook a series of consultations with public, patient and clinical experts/stakeholders (including health services managers and ward-based nurses). These discussions were used to explore views on balancing nursing skill mix (RNs and NAs)

For peer review only - http://bmjopen.bmj.com/site/about/guidelines.xhtml

For peer review only

on the wards and the factors affecting adherence to current vital signs protocols. An independent lay researcher was also part of the research team and advised on public engagement.

RESULTS

Patient admissions, vital signs and staffing levels

Vital sign observations from 138,133 patient admissions (Table 1) were analysed after exclusion criteria were applied (Figure 1). Patients had a median age of 66.6 and Charlson Co-morbidity Index (CCI) of 3 and 80% of admissions were emergencies. The median length of hospital stay was 2.7 days and the cohort had a 4.1% mortality rate.

On average, 16.1% of observations were classified as missed and 30.1% were delayed. Six percent of observations were preceded by a high acuity (NEWS >5) score, of which 44% were classified as missed and 53.5% of observations were delayed. Table 2 shows the rate of delayed and missed observations across the 32 study wards. The rate of missed observations varied substantially between wards, with the highest levels seen on the neuro-rehabilitation and respiratory high care wards (45% and 39% respectively). Mean staffing levels for registered nurses (RNs) were 4.75 hours per patient day (HPPD), with high variation both within and between wards (Supplementary material A1). On average, the within-ward standard deviation of staffing levels was 18% of the mean. Attempts to identify staff groups involved in taking observations were hampered by lack of standard coding. Across all wards an average 15% of observations was recorded as being taken by a NA (16% for low acuity observations, 15% for high acuity observations). However, the lack of standard coding and the large proportion of observations attributed to 'unknown' staff led us to judge these data as unreliable, and so we did not consider them further in the analysis.

Relationship between staffing levels and missed observations

To examine the relationship between missed/delayed observations and staffing levels, we first considered *all* observations. We then performed a sub-group analysis, stratifying observations by acuity (low/medium/high, see Methods). Results for the low and medium acuity sub-groups are in the supplementary material (A2).

All observations

Table 3 (Model A) shows that the rate of missed observations was significantly associated with levels of both RN ($p < 0.0001$) and Nursing Assistant (NA) staffing ($p < 0.001$). The magnitude of the effect (Incidence Rate Ratio, IRR) was greater for nursing assistants (IRR 0.954, 0.949-0.958) than for registered nurses (IRR 0. 983, 0.979-0.987). Measures of

For peer review only - http://bmjopen.bmj.com/site/about/guidelines.xhtml

admissions per registered nurse and the proportion of higher acuity patients were also highly significant ($p < 0.001$). Addition of a linear interaction term between RN and NA (Table 3, Model B) was significant ($p < 0.001$) and improved model fit (AIC 215,974 versus 216,062). Similar relationships were observed for the secondary outcome (delayed observations, see Supplementary material A3).

To further explore the relationship between the two nursing groups and the nature of the interaction, we categorised staffing levels into terciles (Supplementary Table A4). The coefficients from this model were used to visualise the effects of various combinations of staff (Figure 2). Any additional hours from either staff group reduced the rate of missed observations compared to when staffing from both groups was low. Increasing NA staffing from low to medium was associated with substantial reductions in missed observations for all levels of RN staffing. However, increasing NA staffing from medium to high was only associated with a further reduction in missed observations when RN staffing was low, and even then only by a small amount. Conversely, increasing levels of RN staffing was always associated with a reduction in missed care, regardless of the levels of NA staffing.

High acuity observations

Table 4 shows equivalent models (Model A and B) in the sub-group of high acuity observations. In this group, only higher levels of registered nurses were significantly ($p < 0.001$) associated with reductions in the rate of missed observations (IRR 0.982, 95% CI 0.972-0.992). Addition of a linear interaction between RN and NA staffing did not alter the size or significance of the relationship between RN staffing and missed observations, and model fit worsened.

DISCUSSION

This is the first study to examine the relationship between nurse staffing levels and an objective measure of missed care. Furthermore, it is the only study of missed care to focus specifically on vital signs monitoring, which has been implicated in the causal pathway between low staffing and increased mortality [13]. Our results show that higher levels of staffing for both registered nurses and nursing assistants were associated with significantly lower rates of missed observations. There was significant interaction between the effects of RN and HCA staffing levels. Rates of missed or delayed *high* acuity observations were only sensitive to the level of registered nurse staffing with no evidence of interaction between the two staffing levels.

For peer review only - http://bmjopen.bmj.com/site/about/guidelines.xhtml

Monitoring vital signs is a fundamental component of the ‘Chain of Prevention’, a tool that describes the processes required to identify and prevent patient deterioration [21]. Nursing staff clearly play a key role in this process [3,22] and adherence to monitoring protocols provides a plausible mechanism where “missed care” could directly lead to adverse outcomes for patients. Our results are consistent with self-reports of nurses [23] and other studies that have highlighted compliance issues with monitoring [15,24,25].

In the face of ongoing shortages of RNs in many countries, NAs and equivalent staff are increasingly deployed to support RNs to undertake some tasks that would otherwise be undertaken by RNs [26]. With regard to the overall rate of missed observations, there is evidence that NAs may act as labour substitutes for RNs in completing observations in a timely fashion. However, this relationship does not apply for higher acuity patients and their observations. The absence of a main effect for NA staffing, in tandem with no significant interaction effect, suggests that NA staff are neither an effective substitute nor a complementary resource (i.e. enhancing the ability of RN staffing to deliver observations) for timely observation of acutely unwell patients. This seems a surprising finding, given that a key *raison d'être* for NAs is to support the work of RNs [27].

While higher acuity observations are a relatively small proportion of all those taken (6%), patients with a NEWS >5 are at substantially increased risk of dying or experiencing an adverse event such as cardiac arrest within the next 24 hours [28]. Work undertaken by NAs could release RNs to focus on acutely unwell patients (complementarity) but there is some evidence suggesting that NAs are routinely undertaking observations in acutely unwell patients in some settings [24]. Records of the staff group who performed observations in the current study were not fully reliable, although our data are consistent with NAs taking a substantial number of observations for both low and high acuity patients. Absence of substitution and complementarity for NAs in relation to missed nursing care has been demonstrated previously [29] and this finding does serve to emphasise the importance of RNs in ensuring safe care for patients at risk of deterioration. However, the role of the two different staff groups in providing this care merits further investigation.

A key finding of our study is that nurse staffing has a relatively small effect on whether or not vital signs are taken in accordance with protocol. For example, adding one extra hour of RN care per patient per day would result in an absolute reduction of less than 1% in the number of missed high acuity observations. Given the high levels of missed observations (nearly 45% in some wards), it seems clear that most deviations from protocol are attributable to factors other than the number of staff available to make observations. This may be related to the fact that the precise recommended frequencies for monitoring are based on expert opinion [11,12]

For peer review only - http://bmjopen.bmj.com/site/about/guidelines.xhtml

and not supported by direct evidence [30]. Consequently, registered nurses in particular are likely to be exercising their clinical judgement when deciding how often to obtain a set of vital signs. Therefore, we question whether measuring absolute adherence to observation protocols is a valid measure of quality, as it only partially meets the criteria for a good indicator [31,32]. It might also be that other components of the "chain of prevention", such as escalating abnormal observations appropriately, are more sensitive to levels of registered nurses. [21] Nonetheless, reductions in compliance at a ward level may be indicative of deterioration in quality of care and the clinical importance of the small changes we observed are unclear. However, the weak association we observed between staffing levels and compliance suggests that the promise that this might provide a leading indicator for staffing problems that might lead to poor outcomes may not be realised [4].

Conclusion

This is the first study to demonstrate an association between nurse staffing levels and an objective measure of complete and timely care in relation to monitoring patients' vital signs, a key mechanism hypothesised to explain the link between low nurse staffing and increased mortality. Compliance with vital signs monitoring schedules is lower when levels of nurse and nursing assistant staff are lower, although substantial increases in numbers of staff would be required to effect meaningful increase in adherence. It is likely that other factors, such as clinical judgement, are the main drivers of non-adherence.

FUNDING

This project was funded by the NIHR Health Services and Delivery Research Programme (HS&DR 13/114/17). This paper draws on research and data reported in more detail in the NIHR Journal's Library Publication:

P. Griffiths J. Ball, K. Bloor, D. Böhning, J. Briggs, C. Dall'Ora, A. De Iongh, J. Jones, C. Kovacs, A. Maruotti, P. Meredith, D. Prytherch, A. R. Saucedo, O. Redfern, P. Schmidt, N. Sinden and G. Smith. "Nurse staffing levels, missed vital signs and mortality in hospitals: retrospective longitudinal observational study." Health Services and Delivery Research Journal 2018; 6(38).

DISCLAIMER

The views expressed are those of the authors and not necessarily those of the NHS, the NIHR or the Department of Health and Social Care.

For peer review only - http://bmjopen.bmj.com/site/about/guidelines.xhtml

For peer review only

CONTRIBUTIONS OF AUTHORS

PG contributed to the design of the study and acquisition of research funding. PG and OR interpreted the data, and drafted and revised the paper. AM contributed to the design of the study, statistical analysis plan, acquisition of funding and interpretation of data; advised on statistical analysis; contributed to drafting the paper and approved the final manuscript. A R-S and GS contributed to the interpretation of the results and drafting the paper.

Other members of the Missed Care Study Group contributed to the acquisition of funding and/or data, analysis, interpretation of analysis and approval of the paper.

CONFLICT OF INTERESTS STATEMENT

PM, NS and PES are employees of Portsmouth Hospitals NHS Trust (PHT), which had a royalty agreement with The Learning Clinic (TLC) to pay for the use of PHT intellectual property within the Vitalpac product, which expired during the course of this study. DP and GBS are former employees of PHT. PES, and the wives of DP and GBS, held shares in TLC until 2015. JB's research has previously received funding from TLC through a Knowledge Transfer Partnership. PG was an unpaid member of the advisory group for NHS Improvement's work developing improvement resources for safe staffing in adult inpatient wards.

TABLES

**Admissions, N (%)**	138,133 (100%)
***Emergency admissions,* N (%)**	108,865 (79 %)
***Elective,* N (%)**	29 268 (21%)
***Age* median, (range)**	66.6 (16.0-106)
***Charlson Co-morbidity Index (CCI),* median (range)**	3 (0-98)
***First NEWS,* median (range)**	1 (0-19)
Low (NEWS <3) N (%)	102,674 (74%)
Medium (NEWS 3-5) N (%)	27,409 (20%)
High (NEWS >5) N (%)	8,050 (6%)
***Length of stay* in days, median (range)**	2.73 (0.150-933)
***In-hospital mortality* N (%)**	5,662 (4.1%)

Table 1 Admission characteristics for study participants

For peer review only - http://bmjopen.bmj.com/site/about/guidelines.xhtml

WARD	ALL OBSERVATIONS		HIGH ACUITY OBSERVATIONS	
	% Delayed	% Missed	% Delayed	% Missed
SURGICAL: GYNAECOLOGICAL	19.8%	10.4%	38.2%	30.3%
MEDICAL: GASTROENTEROLOGY	42.9%	25.8%	61.0%	52.3%
MEDICAL: CARDIOLOGY/GASTROENTEROLOGY	45.5%	24.7%	62.8%	52.2%
MEDICAL/SURGICAL: CARDIAC HIGH CARE	25.6%	14.0%	48.8%	39.7%
SURGICAL: EMERGENCY ORTHOPAEDIC (SPINAL)	33.2%	19.1%	46.9%	37.4%
MEDICAL: GENERAL	40.9%	22.4%	64.1%	55.1%
MEDICAL: GENERAL	30.8%	14.4%	39.8%	32.0%
SURGICAL: EMERGENCY ORTHOPAEDIC (HEAD INJURY)	24.3%	10.9%	44.4%	35.9%
SURGICAL: ELECTIVE ORTHOPAEDIC	21.3%	11.9%	29.8%	23.3%
SURGICAL: OLDER PEOPLE	31.9%	17.4%	39.7%	30.7%
SURGICAL: GENERAL UROLOGY, VASCULAR, PLASTIC	29.2%	15.0%	45.9%	34.4%
SURGICAL: HEAD & NECK	29.1%	12.9%	53.2%	43.7%
SURGICAL: GENERAL, UPPER GI	21.0%	8.8%	36.3%	27.9%
SURGICAL: GENERAL/COLORECTAL	23.5%	10.6%	44.1%	36.2%
MEDICAL: RESPIRATORY HIGH CARE AND STEP DOWN	52.6%	38.5%	71.8%	64.2%
MEDICAL: RESPIRATORY	47.7%	30.7%	63.5%	53.4%
REHABILITATION: NEURO	61.2%	45.2%	56.2%	47.9%
MEDICAL : OLDER PEOPLE	28.0%	15.2%	51.0%	41.8%
REHABILITATION: STROKE (OLDER PEOPLE)	52.5%	35.8%	53.1%	44.1%
MEDICAL: ACUTE STROKE	40.9%	19.3%	58.7%	49.9%
MEDICAL: RADIOTHERAPY HAEMATOLOGY / ONCOLOGY	24.3%	11.5%	54.8%	44.7%
MEDICAL : OLDER PEOPLE	32.8%	16.4%	56.2%	45.5%
MEDICAL : OLDER PEOPLE	39.6%	19.0%	58.4%	47.3%
MEDICAL : OLDER PEOPLE	38.2%	20.6%	59.7%	49.7%
MEDICAL : OLDER PEOPLE	36.7%	17.2%	60.6%	48.9%
MEDICAL/SURGICAL: ELECTIVE & INVESTIGATIONS	18.7%	8.8%	37.1%	30.5%
MEDICAL: RENAL HIGH CARE	25.6%	13.2%	45.8%	36.4%
MEDICAL: RENAL	21.9%	10.8%	46.5%	38.4%
SURGICAL: RENAL TRANSPLANT	16.3%	7.6%	38.3%	31.5%
MEDICAL: EMERGENCY ADMISSIONS	19.7%	9.1%	50.4%	39.0%
SURGICAL: ADMISSIONS	15.4%	5.6%	39.1%	31.7%
SURGICAL: HIGH CARE	9.8%	5.5%	31.0%	22.4%

Table 2 Percentage of missed and delayed observations for each of the 32 study wards

For peer review only - http://bmjopen.bmj.com/site/about/guidelines.xhtml

MODEL	A			B		
	IRR	95% confidence interval	p-value	IRR	95% confidence interval	p-value
**RN staffing**	0.983	0.979-0.987	< 0.001	0.981	0.977-0.985	< 0.001
**NA staffing**	0.954	0.949-0.958	< 0.001	0.957	0.952-0.961	< 0.001
**Patient turnover**	1.01	1.01-1.01	< 0.001	1.01	1.01-1.02	< 0.001
**Observations in higher acuity patients**	4.83	4.68-4.99	< 0.001	4.8	4.65-4.96	< 0.001
**RN staffing x NA staffing**				1.01	1.01-1.01	< 0.001

Table 3 Mixed-effects Poisson regression: Association between staffing and all missed observations with (A) and without (B) inclusion of a linear interaction term between RN and NA staffing levels. IRR = incidence rate ratio Model A: AIC 215974 BIC 216033 Model B: AIC 216062 BIC 216112

MODEL	A			B		
	IRR	95% confidence interval	p-value	IRR	95% confidence interval	p-value
**RN Staffing**	0.982	0.972-0.992	< 0.001	0.982	0.972-0.992	< 0.001
**NA Staffing**	1	0.990-1.01	0.822	1	0.991-1.01	0.791
**Patient Turnover**	0.997	0.988-1.01	0.59	0.997	0.988-1.01	0.582
**Observations in higher acuity patients**	1.01	0.936-1.09	0.769	1.01	0.937-1.09	0.747
**RN staffing x NA staffing**				0.999	0.994-1.00	0.64

Table 4 Mixed-effects Poisson regression: Association between staffing and high acuity missed observations with (a) and without (b) inclusion of a linear interaction term between RN and NA staffing levels. IRR = incidence rate ratio. Model A: AIC 76747; BIC; 76796 Model B: AIC 76749; BIC 76806

For peer review only

FIGURES

Figure 1 Study flowchart

Figure 2 Partial dependency plots showing interaction effects between levels of registered nurse and nursing assistant (HCA) for all missed observations

For peer review only

For peer review only - http://bmjopen.bmj.com/site/about/guidelines.xhtml

REFERENCES

1 Keogh B. Review into the quality of care and treatment provided by 14 hospital trusts in England: overview report. *NHS* 2013.

2 Page A. *Keeping patients safe: Transforming the work environment of nurses*. National Academies Press 2004.

3 Clarke SP. Failure to rescue: lessons from missed opportunities in care. *Nurs Inq* 2004;**11**:67–71. doi:10.1111/j.1440-1800.2004.00210.x

4 Griffiths P, Recio-Saucedo A, Dall'Ora C, *et al.* The association between nurse staffing and omissions in nursing care: A systematic review. *J Adv Nurs* 2018;**74**:1474–87. doi:10.1111/jan.13564

5 Griffiths P, Ball J, Drennan J, *et al.* Nurse staffing and patient outcomes: Strengths and limitations of the evidence to inform policy and practice. A review and discussion paper based on evidence reviewed for the National Institute for Health and Care Excellence Safe Staffing guideline develo. *Int J Nurs Stud* 2016;**63**:213–25. doi:10.1016/j.ijnurstu.2016.03.012

6 Kalisch BJ, Landstrom GL, Hinshaw AS. Missed nursing care: a concept analysis. *J Adv Nurs* 2009;**65**:1509–17. doi:10.1111/j.1365-2648.2009.05027.x

7 Schubert M, Glass TR, Clarke SP, *et al.* Validation of the Basel Extent of Rationing of Nursing Care Instrument. *Nurs Res* 2007;**56**:416–24. doi:10.1097/01.NNR.0000299853.52429.62

8 Lucero RJ, Lake ET, Aiken LH. Nursing care quality and adverse events in US hospitals. *J Clin Nurs* 2010;**19**:2185–95. doi:10.1111/j.1365-2702.2010.03250.x

9 Ausserhofer D, Zander B, Busse R, *et al.* Prevalence, patterns and predictors of nursing care left undone in European hospitals: results from the multicountry cross-sectional RN4CAST study. *BMJ Qual Saf* 2014;**23**:126–35. doi:10.1136/bmjqs-2013-002318

10 Ball JE, Bruyneel L, Aiken LH, *et al.* Post-operative mortality, missed care and nurse staffing in nine countries: A cross-sectional study. *Int J Nurs Stud* 2018;**78**:10–5. doi:10.1016/j.ijnurstu.2017.08.004

11 National Institute for Health and Care Excellence. Acutely ill patients in hospital: Recognition of and response to acute illness in adults in hospital. NICE guideline (50). London: 2007.

For peer review only - http://bmjopen.bmj.com/site/about/guidelines.xhtml

12 Royal College of Physicians (London). National Early Warning Score (NEWS): Standardising the assessment of acute illness severity in the NHS. Report of a working party. 2012.

13 National Patient Safety Agency. Recognising and responding appropriately to early signs of deterioration in hospitalised patients. 2007. doi:accessed 03.04.14

14 Smith GB, Prytherch DR, Schmidt PE, *et al.* A review, and performance evaluation, of single-parameter 'track and trigger' systems. *Resuscitation* 2008;**79**:11–21. doi:10.1016/j.resuscitation.2008.05.004

15 Hands C, Reid E, Meredith P, *et al.* Patterns in the recording of vital signs and early warning scores: compliance with a clinical escalation protocol. *BMJ Qual Saf* 2013;**22**:719–26. doi:10.1136/bmjqs-2013-001954

16 Recio-Saucedo A, Maruotti A, Griffiths P, *et al.* Relationships between healthcare staff characteristics and the conduct of vital signs observations at night: Results of a survey and factor analysis. *Nurs Open* 2018;**5**:621–33. doi:10.1002/nop2.179

17 Antonakis J, Bendahan S, Jacquart P, *et al.* On making causal claims: A review and recommendations. *Leadersh Q* 2010;**21**:1086–120. doi:10.1016/j.leaqua.2010.10.010

18 Smith GB, Prytherch DR, Schmidt P, *et al.* Hospital-wide physiological surveillance-A new approach to the early identification and management of the sick patient. *Resuscitation* 2006;**71**:19–28. doi:10.1016/j.resuscitation.2006.03.008

19 R Core Team. R: A Language and Environment for Statistical Computing. 2017.

20 Bates D, Mächler M, Bolker B, *et al.* Fitting Linear Mixed-Effects Models Using lme4. *J Stat Softw* 2015;**67**. doi:10.18637/jss.v067.i01

21 Smith GB. In-hospital cardiac arrest: Is it time for an in-hospital 'chain of prevention'? *Resuscitation* 2010;**81**:1209–11. doi:10.1016/j.resuscitation.2010.04.017

22 Griffiths P, Jones S, Bottle A. Is "failure to rescue" derived from administrative data in England a nurse sensitive patient safety indicator for surgical care? Observational study. *Int J Nurs Stud* 2013;**50**:292–300. doi:10.1016/j.ijnurstu.2012.10.016

23 Zander B, Dobler L, Bäumler M, *et al.* Implizite Rationierung von Pflegeleistungen in deutschen Akutkrankenhäusern – Ergebnisse der internationalen Pflegestudie RN4Cast. *Das Gesundheitswes* 2014;**76**:727–34. doi:10.1055/s-0033-1364016

24 JAMES J, BUTLER-WILLIAMS C, HUNT J, *et al.* Vital signs for vital people: an exploratory study into the role of the Healthcare Assistant in recognising, recording and

For peer review only - http://bmjopen.bmj.com/site/about/guidelines.xhtml

responding to the acutely ill patient in the general ward setting. *J Nurs Manag* 2010;**18**:548–55. doi:10.1111/j.1365-2834.2010.01086.x

25 Griffiths P, Saucedo AR, Schmidt P, *et al.* Vital signs monitoring in hospitals at night. *Nurs Times* 2015;**111**:16–7.

26 Duffield CM, Twigg DE, Pugh JD, *et al.* The Use of Unregulated Staff: Time for Regulation? *Policy, Polit Nurs Pract* 2014;**15**:42–8. doi:10.1177/1527154414529337

27 Koyner JL, Carey KA, Edelson DP, *et al.* The Development of a Machine Learning Inpatient Acute Kidney Injury Prediction Model*. *Crit Care Med* 2018;**46**:1070–7. doi:10.1097/CCM.0000000000003123

28 Smith GB, Prytherch DR, Meredith P, *et al.* The ability of the National Early Warning Score (NEWS) to discriminate patients at risk of early cardiac arrest, unanticipated intensive care unit admission, and death. *Resuscitation* 2013;**84**:465–70. doi:10.1016/j.resuscitation.2012.12.016

29 Ball JE, Murrells T, Rafferty AM, *et al.* 'Care left undone' during nursing shifts: associations with workload and perceived quality of care. *BMJ Qual Saf* 2014;**23**:116–25. doi:10.1136/bmjqs-2012-001767

30 Smith GB, Recio-Saucedo A, Griffiths P. The measurement frequency and completeness of vital signs in general hospital wards: An evidence free zone? *Int J Nurs Stud* Published Online First: July 2017. doi:10.1016/j.ijnurstu.2017.07.001

31 Mainz J. Developing evidence-based clinical indicators: a state of the art methods primer. *Int J Qual Heal Care* 2003;**15**:5i – 11. doi:10.1093/intqhc/mzg084

32 Mainz J. Defining and classifying clinical indicators for quality improvement. *Int J Qual Heal Care* 2003;**15**:523–30. doi:10.1093/intqhc/mzg081

For peer review only

For peer review only - http://bmjopen.bmj.com/site/about/guidelines.xhtml

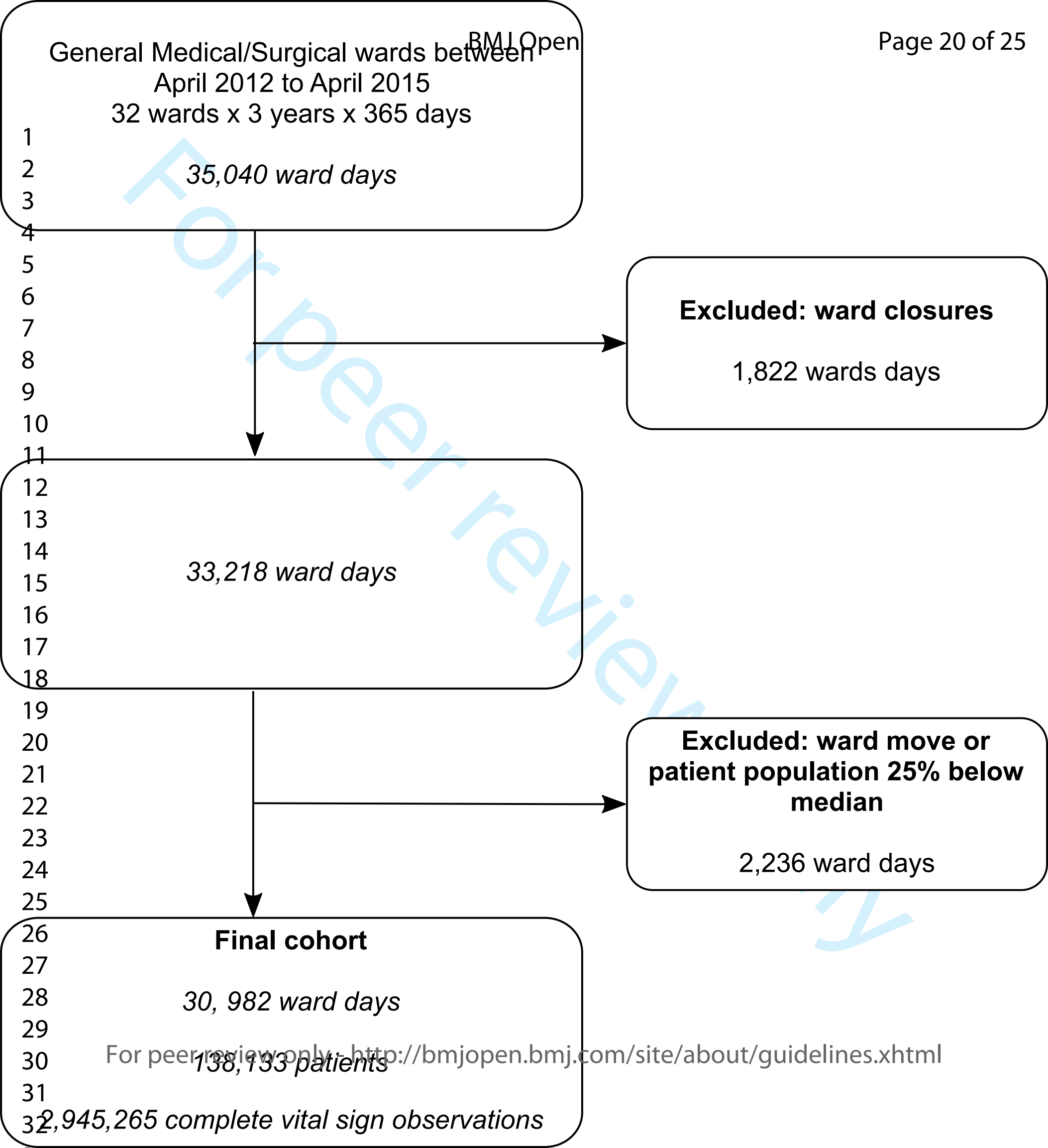

For peer review only

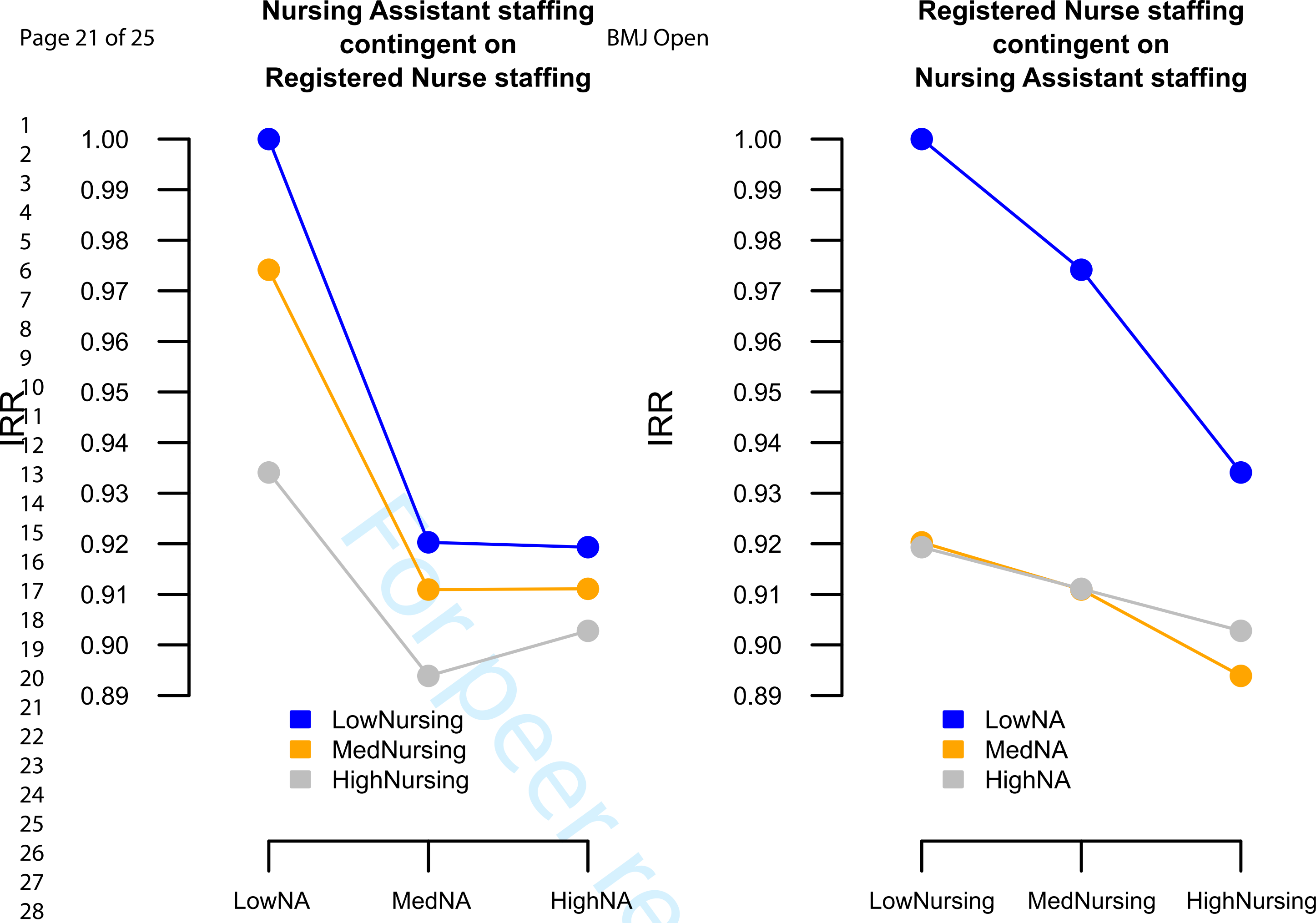

For peer review only

SUPPLEMENTARY MATERIAL

A1 Mean staffing per ward

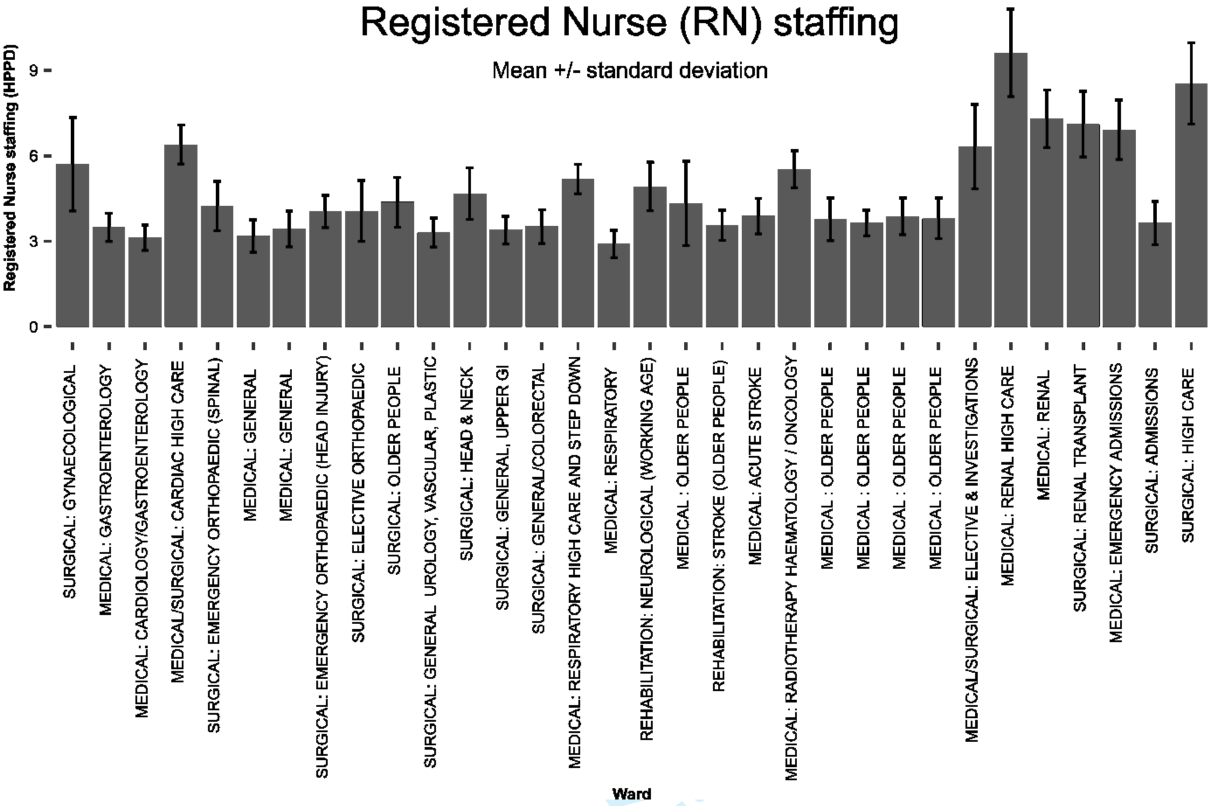

A2 Missed observations (low and medium acuity)

The tables below show the relationship of staffing levels with respect to the primary outcome (missed observations) for low and medium acuity observations.

	IRR	95% confidence Interval	p-value
**RN staffing**	0.98	0.973-0.986	< 0.001
**NA staffing**	0.933	0.926-0.939	< 0.001
**Patient turnover**	1.04	1.03-1.04	< 0.001
**Higher acuity patients**	2.03	1.93-2.14	< 0.001
**RN staffing x NA staffing**	1.02	1.02-1.02	< 0.001

Table A2a Low acuity

	IRR	95% confidence Interval	p-value
**RN staffing**	0.977	0.971-0.984	< 0.001
**NA staffing**	0.964	0.957-0.971	< 0.001
**Patient turnover**	0.989	0.984-0.995	< 0.001
**Higher acuity patients**	0.641	0.609-0.676	< 0.001

iew only

**RN staffing x NA staffing**	1.01	1.00-1.01	< 0.001

Table A2b Medium acuity

A3 Delayed observations

The tables below show the relationship of staffing levels with respect to the secondary outcome (delayed observations).

	IRR	95% confidence Interval	p-value
**RN staffing**	0.984	0.981-0.987	< 0.001
**NA staffing**	0.98	0.976-0.983	< 0.001
**Patient turnover**	1	1.00-1.01	0.0186
**Higher acuity patients**	2.23	2.18-2.28	0
**RN staffing x NA staffing**	1.01	1.00-1.01	< 0.001

Table A3a All observations

	IRR	95% confidence Interval	p-value
**RN staffing**	0.987	0.978-0.996	0.0043
**NA staffing**	1	0.993-1.01	0.55
**Patient turnover**	0.996	0.987-1.00	0.38
**Higher acuity patients**	1.03	0.960-1.10	0.415
**RN staffing x NA staffing**	1	0.995-1.00	0.943

Table A3b High acuity observations

A4 Missed observations interactions

The table below shows the model used to explore interactions between NA and RN staffing groups (see Figure 2 in main manuscript).

	IRR	95% confidence interval	p-value
**RN staffing Q2**	0.974	0.963-0.985	p < 0.001
**RN staffing Q3**	0.934	0.923-0.946	p < 0.001
**NA staffing Q2**	0.92	0.910-0.931	p < 0.001
**NA staffing Q3**	0.919	0.908-0.931	p < 0.001
**Patient turnover**	1.01	1.01-1.02	p < 0.001
**Higher acuity patients**	4.83	4.68-4.99	0
**RN staffing Q2 x NA staffing Q2**	1.02	0.999-1.03	0.0603
**RN staffing Q3 x NA staffing Q2**	1.04	1.02-1.06	p < 0.001
**RN staffing Q2 x NA staffing Q3**	1.02	1.00-1.04	0.0525
**RN staffing Q3 x NA staffing Q3**	1.05	1.03-1.07	p < 0.001

Table A4 Mixed-effects Poisson regression with staffing variables modelled as tertiles: Association between staffing and all missed observations AIC: 215,999 BIC: 216,099

For peer review only

2STROBE 2007 (v4) Statement—Checklist of items that should be included in reports of *cohort studies*

Section/Topic	Item #	Recommendation	Reported on page #
**Title and abstract**	1	(*a*) Indicate the study's design with a commonly used term in the title or the abstract	2
		(*b*) Provide in the abstract an informative and balanced summary of what was done and what was found	2
**Introduction**			
Background/rationale	2	Explain the scientific background and rationale for the investigation being reported	4-5
Objectives	3	State specific objectives, including any prespecified hypotheses	5
**Methods**			
Study design	4	Present key elements of study design early in the paper	5
Setting	5	Describe the setting, locations, and relevant dates, including periods of recruitment, exposure, follow-up, and data collection	5
Participants	6	(*a*) Give the eligibility criteria, and the sources and methods of selection of participants. Describe methods of follow-up	5-6
		(*b*) For matched studies, give matching criteria and number of exposed and unexposed	N/A
Variables	7	Clearly define all outcomes, exposures, predictors, potential confounders, and effect modifiers. Give diagnostic criteria, if applicable	6-7
Data sources/ measurement	8*	For each variable of interest, give sources of data and details of methods of assessment (measurement). Describe comparability of assessment methods if there is more than one group	*6-7*
Bias	9	Describe any efforts to address potential sources of bias	5
Study size	10	Explain how the study size was arrived at	6
Quantitative variables	11	Explain how quantitative variables were handled in the analyses. If applicable, describe which groupings were chosen and why	6,7
Statistical methods	12	(*a*) Describe all statistical methods, including those used to control for confounding	7
		(*b*) Describe any methods used to examine subgroups and interactions	7
		(*c*) Explain how missing data were addressed	N/A
		(*d*) If applicable, explain how loss to follow-up was addressed	N/A
		(*e*) Describe any sensitivity analyses	N/A
**Results**			

For peer review only

Participants	13*	(a) Report numbers of individuals at each stage of study—eg numbers potentially eligible, examined for eligibility, confirmed eligible, included in the study, completing follow-up, and analysed	Figure 1
		(b) Give reasons for non-participation at each stage	Figure 1
		(c) Consider use of a flow diagram	Figure 1
Descriptive data	14*	(a) Give characteristics of study participants (eg demographic, clinical, social) and information on exposures and potential confounders	8, Table 1
		(b) Indicate number of participants with missing data for each variable of interest	N/A
		(c) Summarise follow-up time (eg, average and total amount)	Table 1
Outcome data	15*	Report numbers of outcome events or summary measures over time	8, Table 1, Table 2
Main results	16	(*a*) Give unadjusted estimates and, if applicable, confounder-adjusted estimates and their precision (eg, 95% confidence interval). Make clear which confounders were adjusted for and why they were included	Table 3
		(*b*) Report category boundaries when continuous variables were categorized	Figure 2, Supp A2,
		(*c*) If relevant, consider translating estimates of relative risk into absolute risk for a meaningful time period	N/A
Other analyses	17	Report other analyses done—eg analyses of subgroups and interactions, and sensitivity analyses	9, Supp file
**Discussion**			
Key results	18	Summarise key results with reference to study objectives	9
**Limitations**			
Interpretation	20	Give a cautious overall interpretation of results considering objectives, limitations, multiplicity of analyses, results from similar studies, and other relevant evidence	9-11
Generalisability	21	Discuss the generalisability (external validity) of the study results	10
**Other information**			
Funding	22	Give the source of funding and the role of the funders for the present study and, if applicable, for the original study on which the present article is based	11

*Give information separately for cases and controls in case-control studies and, if applicable, for exposed and unexposed groups in cohort and cross-sectional studies.

Note: An Explanation and Elaboration article discusses each checklist item and gives methodological background and published examples of transparent reporting. The STROBE checklist is best used in conjunction with this article (freely available on the Web sites of PLoS Medicine at http://www.plosmedicine.org/, Annals of Internal Medicine at http://www.annals.org/, and Epidemiology at http://www.epidem.com/). Information on the STROBE Initiative is available at www.strobe-statement.org.

For peer review only

The association between nurse staffing levels and the timeliness of vital signs monitoring: a retrospective observational study in the UK

Journal:	*BMJ Open*
Manuscript ID	bmjopen-2019-032157.R1
Article Type:	Original research
Date Submitted by the Author:	15-Aug-2019
Complete List of Authors:	Redfern, Oliver; Oxford University, Nuffield Department of Clinical Neurosciences<br>Griffiths, Peter; University of Southampton<br>Maruotti, Antonello; Libera Universita Maria Santissima Assunta, Dipartimento di Scienze Economiche<br>Recio-Saucedo, Alejandra; University of Southampton<br>Smith, Gary; University of Bournemouth, School of Health and Social Care
**Primary Subject Heading**:	Health informatics
Secondary Subject Heading:	Health policy, Medical management
Keywords:	nursing staff, vital signs, patient deterioration, Retrospective study, Observational Study

SCHOLARONE™ Manuscripts

The association between nurse staffing levels and the timeliness of vital signs monitoring: a retrospective observational study in the UK

AUTHORS

*Oliver Redfern[e,i]

*†Peter Griffiths[a,b,c]

Antonello Maruotti[a, j]

Alejandra Recio-Saucedo[a,b]

Gary Smith[h]

The Missed Care Study Group [+]

[+] The Missed Care Study Group comprises Peter Griffiths (a, b, c, g), Jane Ball (a, b, c), Karen Bloor (d), Dankmar Böhning (a), Jim Briggs (e, g), Chiara Dall'Ora (a, b), Anya De Iongh (f), Jeremy Jones (a), Caroline Kovacs (e), Antonello Maruotti (a), Paul Meredith (b, g), Alejandra Recio-Saucedo (a, b), David Prytherch (e, g), Oliver Redfern (e, i), Paul Schmidt (b, g), Nicky Sinden (g), and Gary Smith (h).

a. University of Southampton, Health Sciences
b. National Institute for Health Research Collaboration for Applied Health Research and Care (Wessex)
c. Karolinska Instutet, Department of Learning, Informatics, Management and Ethics
d. University of York, Health Sciences
e. University of Portsmouth, Centre for Healthcare Modelling and Informatics
f. Independent lay researcher
g. Portsmouth Hospitals NHS Trust, Clinical Outcomes Research Group

For peer review only - http://bmjopen.bmj.com/site/about/guidelines.xhtml

h. Bournemouth University, Faculty of Health and Social Sciences

i. Nuffield Department of Clinical Neurosciences, University of Oxford

j. LUMSA Universita Maria SS. Assunta di Roma, Department of economic science, politics and modern languages

* To be considered as joint first authors

† **Corresponding author**

Professor Peter Griffiths

Chair of Health Services Research

University of Southampton

Room 3021, Building 67, Highfield Campus, Southampton SO17 1BJ

Tel. +44 2380 597877

Email: peter.griffiths@soton.ac.uk

KEYWORDS

Nursing staff, vital signs, patient deterioration, retrospective study, observational study

For peer review only - http://bmjopen.bmj.com/site/about/guidelines.xhtml

ABSTRACT

Objectives

Omissions and delays in delivering nursing care are widely-reported consequences of staffing shortages, with potentially serious consequences. However, studies so far have relied exclusively on nurse self-reporting. Monitoring vital signs is a key part of nursing work and electronic recording provides an opportunity to objectively measure delays in care. This study aimed to determine the association between registered nurse (RN) and nursing assistant (NA) staffing levels and adherence to a vital signs monitoring protocol.

Design

Retrospective observational study.

Setting

32 medical and surgical wards in an acute general hospital in England.

Participants

538,238 nursing shifts taken over 30,982 ward days.

Primary and secondary outcome measures

Vital sign observations were scheduled according to a protocol based on the National Early Warning Score (NEWS). The primary outcome was the daily rate of missed vital signs (overdue by ≥67% of the expected time to next observation). The secondary outcome was the daily rate of late vital signs observations (overdue by ≥33%). We undertook sub-group analysis by stratifying observations into low, medium and high acuity using the NEWS score.

Results

Late and missed observations were frequent, particularly in high acuity patients (median = 44%). Higher levels of registered nurse staffing, measured in hours per patient per day (HPPD), were associated with a lower rate of missed observations in all (Incident Rate ratio [IRR] 0.980, 95% Confidence Interval [CI] 0.973-0.986) and high acuity patients (0.982, 0.972-0.992). However, levels of nursing assistant staffing were only associated with the daily rate (0.933, CI 0.926-0.939) of all missed observations.

Conclusions

Adherence to vital sign monitoring protocols is sensitive to levels of nurse and nursing assistant staffing, although high acuity observations appeared unaffected by levels of nursing assistants. We demonstrate that objectively measured omissions in care are related to nurse staffing levels, although the absolute effects are small.

For peer review only - http://bmjopen.bmj.com/site/about/guidelines.xhtml

STUDY REGISTRATION

The data and analyses presented here were part of the larger Missed Care study (ISRCTN registration: 17930973).

STRENGTHS AND LIMITATIONS

- This was a large retrospective cohort study exploring the association between levels of nursing staff in an acute hospital and adherence to a vital signs monitoring protocol.
- In contrast to previous studies that relied on nursing staff self-reporting missed care, we used an objective measure derived from electronically recorded vital signs.
- This study was limited to a single hospital and we were only able to partially adjust for other factors that might affect protocol adherence.

For peer review only

INTRODUCTION

Reports from around the world have highlighted poor nursing care as a cause of avoidable harm [1–3]. Perhaps unsurprisingly, there is mounting evidence that quality of care deteriorates when wards are understaffed [4], yet the extent to which low staffing leads directly to worse outcomes for patients remains in dispute [5]. A number of studies have explored whether nursing work that is delayed or left incomplete (often referred to as “missed care” [6]) provides a plausible causal mechanism leading to worse patient outcomes, as nurses do not have capacity to deliver all required care when staffing levels are inadequate [4,7]. However, details of nursing activities are not always routinely collected or recorded in standard formats, or in systems that can be easily interrogated, by health care providers. Therefore, it is difficult to measure the timing of care or the extent to which care is delivered [8]. Consequently, the evidence supporting an association between missed care and staffing levels is largely based on nurses’ self-reports [4,9,10].

Recording patients’ vital signs is a fundamental aspect of nursing work, and a key component of patient surveillance: infrequent monitoring can cause signs of clinical deterioration to be missed, leading to delays in administering remedial treatment [3,11,12]. A Europe-wide cross-sectional study (RN4CAST) found that 27% of nursing staff reported missing at least some necessary patient surveillance on their last shift [9]. The failure to properly observe and record vital signs observations has been noted as a factor in inquiries into the cause of preventable death in hospital patients [13].

In response to the increasing recognition that monitoring vital signs is sub-optimal, a number of protocols which define observation schedules have been developed and implemented. For example, on general medical and surgical wards, UK guidelines recommend that the frequency of monitoring is directed by the National Early Warning Score (NEWS) [12]. This is a score that provides a composite measure of patients’ physiological abnormalities, based on vital signs measurements: in general, the higher the score, the more frequently patients should be observed. Internationally, a range of similar early warning or escalation systems are used to guide the observation and escalation of care for at-risk patients [14]. However, retrospective studies have shown, at best, partial adherence to monitoring protocols, particularly at night [15,16].

Inadequate staffing is one possible explanation for this lack of adherence, as it may reduce nurses’ capacity to monitor and intervene to prevent deterioration. This could be one explanation for the association between low nurse staffing levels and increased mortality, which has been demonstrated in many studies worldwide [5]. Yet, existing studies linking low staffing to missed care have exclusively used self-report by nurses derived from cross

For peer review only - http://bmjopen.bmj.com/site/about/guidelines.xhtml

sectional surveys [4]. Such studies suffer a number of limitations, including common-method bias, because all variables are derived from the same self-report survey [17].

In this retrospective observational study of an acute hospital in England, we used routinely collected records of vital signs and other clinical and administrative data, including the electronic rostering database, to investigate whether adherence to the hospital's vital signs monitoring protocol was sensitive to the daily levels of nursing staff.

METHODS

Study Design and Setting

This was a retrospective longitudinal observational study of 32 wards in a large acute hospital in the South of England over three years (April 2012 to March 2015).

Data sources and linkage

Sources

This study combined data from four sources. Information on patients (admissions, ward transfers) was obtained from the Patient Administration System (PAS), allowing us to calculate bed occupancy and the number of admissions to each ward. Vital signs observations were obtained from the Vitalpac™ system [18]. Data items were: anonymised admission identifier, time of observation, National Early Warning Score (NEWS) [12], time to next observation.

Levels of nurse staffing were derived from two source databases. For standard contractual shifts, we extracted data from an electronic rostering system, detailing the date, location, number of hours and grade of each nurse for every shift. The second source was a similar database recording all bank (extra contractual work by staff employed by the hospital) and agency (staff employed through an external agency) shifts.

In total we identified 538,238 shifts worked over the study period by either registered nurses (RNs; fully qualified nurses on the Nursing and Midwifery Council Register with university diploma or degree level qualification or equivalent) or NAs (nursing assistant personnel with no formal training requirements or registration, typically employed in roles described as health care assistants in NHS pay bands 2-4). We did not have access to data on shifts undertaken by student nurses. However, they are considered supernumerary for the purposes for staff allocation.

Linkage

Nursing shifts worked on each day of the study were linked to vital signs observations and admission data (from PAS) using ward location identifiers and time stamps. For each of the

For peer review only - http://bmjopen.bmj.com/site/about/guidelines.xhtml

32 wards, we calculated daily patient and staffing levels. From a theoretical maximum of 35,040 ward days (365 days x 3 years x 32 wards) there were 1,822 (5.2%) ward days where one or more of the study wards was closed and 2,236 (6.4%) ward days where we could not properly match patient records and staffing. This was generally when wards relocated and nursing shifts were still recorded in the old location for a while. Additionally, periods when wards opened, closed or transferred were often associated with unusual values for patient numbers or staff/patient ratios due to low patient census or delayed recording of staff transfers to the new unit. Therefore, we excluded all ward days where the patient census fell below 25% of the ward median. We were unable to link e-roster to the staff taking the observations as no standard identifier was available although we attempted to identify the grade of staff taking the observations using a descriptive field in the Vitalpac™ system.

Outcomes

A total of 2,864,975 complete sets of vital signs were available for analysis. The primary outcome of the study was *missed* vital signs observations. The secondary outcome was *delayed* observations.

Both outcomes were calculated with reference to the hospital's vital signs monitoring protocol. The protocol is based on the National Early Warning Score (NEWS)[12], where the level of derangement in vital signs (the NEWS value) is aggregated into a single integer. This is then used to determine when the patient should next be observed – in general, higher scores prompt more frequent observation. For example, if the NEWS value is 2, the patient should be observed at least every 6 hours. Patients with the lowest score (NEWS = 0) are observed every 12 hours and those with higher scores more frequently.

We defined a vital signs observation as *missed* if overdue by more than 67% of the expected time to next observation determined by the previous NEWS value. Similarly, an observation was *delayed* if overdue by more than 33% of the expected time to next observation determined by the previous NEWS value. For example, if the next observation was due in 60 minutes, it was classified as *delayed* if taken > 80 minutes after the previous observation and *missed* if taken > 100 minutes later.

For sub-group analyses, missed/delayed observations were further stratified in acuity categories according to the previous NEWS value. The study hospital's monitoring protocol (see supplementary material A1) was used to define the following groups:

- Low: where previous NEWS value < 3
- Medium: where previous NEWS value was between 3-5
- High: where previous NEWS value > 5

For peer review only - http://bmjopen.bmj.com/site/about/guidelines.xhtml

Exposures

For each study day on each ward, we calculated the average staffing levels in Hours per Patient Day (HPPD) for both RNs and NAs. HPPD was calculated by dividing the total number of nursing hours worked by the daily bed occupancy (for that ward). Daily bed occupancy was calculated from the PAS database where a value of one indicates a single bed being occupied continuously for one day. A HPPD of 24 indicates one-to-one nursing.

To account for variations in other aspects of nursing workload, we derived variables to quantify admission rates ("patient turnover") and the proportion of observations that were for patients requiring 4-hourly or more frequent observation on each day of the study (i.e. patient with NEWS value ≥3, "higher acuity"). Patient turnover was calculated by dividing total daily RN staffing (in days) by the number of new admissions.

Statistical methods

We chose mixed-effects Poisson regression as our modelling framework to examine the relationship between missed/delayed observations and staffing. Random effects terms were introduced for each ward. All other co-variates were added as fixed effects in the models. Where not otherwise stated, all summary measures are reported using median and interquartile range. All analyses were undertaken using the R statistical environment v3.5 [19] and mixed-effects models were fit using the lme4 package [20].

By modelling the effect of each staff group separately, we considered the extent to which the labour inputs from one group might substitute for the other. Additionally, we tested for potential that NAs acted as labour complements, enhancing the effectiveness of RNs by adding interaction terms to each model. We assessed whether these terms improved model fit by examining the Akaike information criterion (AIC) and Bayesian information criterion (BIC).

Ethical approval

The study was approved by the National Research Ethics Service. East Midlands – Northampton Committee Ref: 15/EM/0099. All identifiable information for patients and staff was removed at source by PM. Internal identifiers were anonymised prior to transfer to the research database. Consequently, it was not possible for the research team to identify participants in the study.

Patient and Public Involvement

As part of the parent study, we undertook a series of consultations with public, patient and clinical experts/stakeholders (including health services managers and ward-based nurses). These discussions were used to explore views on balancing nursing skill mix (RNs and NAs)

For peer review only - http://bmjopen.bmj.com/site/about/guidelines.xhtml

on the wards and the factors affecting adherence to current vital signs protocols. An independent lay researcher was also part of the research team and advised on public engagement.

RESULTS

Patient admissions, vital signs and staffing levels

Vital sign observations from 138,133 patient admissions (Table 1) were analysed after exclusion criteria were applied (Supplementary Figure A2). Patients had a median age of 66.6 and Charlson Co-morbidity Index (CCI) of 3 and 79% of admissions were emergencies. The median length of hospital stay was 2.7 days and the cohort had a 4.1% mortality rate.

On average, 17.1% of all observations across the study wards were classified as missed and 31.3% were delayed. Six percent of observations were preceded by a high acuity (NEWS >5) score, of which 44% were classified as missed and 53.5% of observations were delayed. Table 2 shows the rate of delayed and missed observations across the 32 study wards. The rate of missed observations varied substantially between wards, with the highest levels seen on the neuro-rehabilitation and respiratory high care wards (45% and 39% respectively). Mean staffing levels for registered nurses (RNs) were 4.75 hours per patient day (HPPD), with high variation both within and between wards (Supplementary material A3). On average, the within-ward standard deviation of staffing levels was 18% of the mean. Attempts to identify staff groups involved in taking observations were hampered by lack of standard coding. Across all wards an average 15% of observations was recorded as being taken by a NA (16% for low acuity observations, 15% for high acuity observations). However, the lack of standard coding and the large proportion of observations attributed to 'unknown' staff led us to judge these data as unreliable, and so we did not consider them further in the analysis.

Relationship between staffing levels and missed observations

To examine the relationship between missed/delayed observations and staffing levels, we first considered *all* observations. We then performed a sub-group analysis, stratifying observations by acuity (low/medium/high, see Methods). Results for the low and medium acuity sub-groups are in the supplementary material (A4).

All observations

Table 3 (Model A) shows the relationship between staffing levels and measure of daily nursing workload with the rate of all missed observations. The rate of missed observations was significantly associated with levels of both RN ($p < 0.0001$) and Nursing Assistant (NA) staffing ($p < 0.001$). The magnitude of the effect (Incidence Rate Ratio, IRR) was greater for nursing

For peer review only - http://bmjopen.bmj.com/site/about/guidelines.xhtml

assistants (IRR 0.954, 0.949-0.958) than for registered nurses (IRR 0. 983, 0.979-0.987). Measures of admissions per registered nurse and the proportion of higher acuity patients were also highly significant ($p < 0.001$). We introduced a linear interaction term between RN and NA staffing levels into the model, as we hypothesised that levels of one staffing group may be dependent on the effect of the other. Addition of this interaction term (Table 3, Model B) was significant ($p < 0.001$) and improved model fit (AIC 215,974 versus 216,062). Similar relationships were observed for the secondary outcome (delayed observations, see Supplementary material A5).

To further explore the relationship between the two nursing groups and the nature of the interaction, we categorised staffing levels into tertiles (Supplementary Table A6). The coefficients from this model were used to visualise the effects of various combinations of staff (Figure 1). Any additional hours from either staff group reduced the rate of missed observations compared to when staffing from both groups was low. Increasing NA staffing from low to medium was associated with substantial reductions in missed observations for all levels of RN staffing. However, increasing NA staffing from medium to high was only associated with a further reduction in missed observations when RN staffing was low, and even then only by a small amount. Conversely, increasing levels of RN staffing was always associated with a reduction in missed care, regardless of the levels of NA staffing.

High acuity observations

Table 4 shows equivalent models (Model A and B) in the sub-group of high acuity observations. In this group, only higher levels of registered nurses were significantly ($p < 0.001$) associated with reductions in the rate of missed observations (IRR 0.982, 95% CI 0.972-0.992). Addition of a linear interaction between RN and NA staffing did not alter the size or significance of the relationship between RN staffing and missed observations, and model fit worsened.

DISCUSSION

Main findings

This is the first study to examine the relationship between nurse staffing levels and an objective measure of missed care. Furthermore, it is the only study of missed care to focus specifically on vital signs monitoring, which has been implicated in the causal pathway between low staffing and increased mortality [13]. Our results show that higher levels of staffing for both registered nurses and nursing assistants were associated with significantly lower rates of missed observations. There was significant interaction between the effects of

For peer review only - http://bmjopen.bmj.com/site/about/guidelines.xhtml

RN and NA staffing levels. Rates of missed or delayed *high* acuity observations were only sensitive to the level of registered nurse staffing with no evidence of interaction between the two staffing levels.

Monitoring vital signs is a fundamental component of the ‘Chain of Prevention’, a tool that describes the processes required to identify and prevent patient deterioration [21]. Nursing staff clearly play a key role in this process [3,22] and adherence to monitoring protocols provides a plausible mechanism where “missed care” could directly lead to adverse outcomes for patients. Our results are consistent with self-reports of nurses [23] and other studies that have highlighted compliance issues with monitoring [15,24,25].

In the face of ongoing shortages of RNs in many countries, NAs and equivalent staff are increasingly deployed to support RNs to undertake some tasks that would otherwise be undertaken by RNs [26]. With regard to the overall rate of missed observations, there is evidence that NAs may act as labour substitutes for RNs in completing observations in a timely fashion. However, this relationship does not apply for higher acuity patients and their observations. The absence of a main effect for NA staffing, in tandem with no significant interaction effect, suggests that NA staff are neither an effective substitute nor a complementary resource (i.e. enhancing the ability of RN staffing to deliver observations) for timely observation of acutely unwell patients. This seems a surprising finding, given that a key *raison d'être* for NAs is to support the work of RNs [27].

While higher acuity observations are a relatively small proportion of all those taken (6%), patients with a NEWS >5 are at substantially increased risk of dying or experiencing an adverse event such as cardiac arrest within the next 24 hours [28]. Work undertaken by NAs could release RNs to focus on acutely unwell patients (complementarity) but there is some evidence suggesting that NAs are routinely undertaking observations in acutely unwell patients in some settings [24]. Records of the staff group who performed observations in the current study were not fully reliable, although our data are consistent with NAs taking a substantial number of observations for both low and high acuity patients. Absence of substitution and complementarity for NAs in relation to missed nursing care has been demonstrated previously [29] and this finding does serve to emphasise the importance of RNs in ensuring safe care for patients at risk of deterioration. However, the role of the two different staff groups in providing this care merits further investigation.

A key finding of our study is that nurse staffing has a relatively small effect on whether or not vital signs are taken in accordance with protocol. For example, adding one extra hour of RN care per patient per day would result in an absolute reduction of less than 1% in the number of missed high acuity observations. Given the high levels of missed observations (nearly 45%

For peer review only - http://bmjopen.bmj.com/site/about/guidelines.xhtml

in some wards), it seems clear that most deviations from protocol are attributable to factors other than the number of staff available to make observations. This may be related to the fact that the precise recommended frequencies for monitoring are based on expert opinion [11,12] and not supported by direct evidence [30]. Consequently, registered nurses in particular are likely to be exercising their clinical judgement when deciding how often to obtain a set of vital signs. Therefore, we question whether measuring absolute adherence to observation protocols is a valid measure of quality, as it only partially meets the criteria for a good indicator [31,32]. It might also be that other components of the "chain of prevention", such as escalating abnormal observations appropriately, are more sensitive to levels of registered nurses. [21] Nonetheless, reductions in compliance at a ward level may be indicative of deterioration in quality of care and the clinical importance of the small changes we observed are unclear. However, the weak association we observed between staffing levels and compliance suggests that the promise that this might provide a leading indicator for staffing problems that might lead to poor outcomes may not be realised [4].

Limitations

The main limitation of our study is that it relies on observational data from a single acute hospital. We can therefore only demonstrate an association, rather than causal link, between adherence to monitoring protocols and levels of nursing staff. However, previous studies have relied on cross-sectional designs where levels of missed care and staffing are derived from staff surveys [23]. Our study design eliminated a number of plausible alternative explanations for the association, including common methods bias.

While using routinely-collected vitals to quantify adherence to monitoring protocols gives a more objective measure of missed care, it is not without its own limitations. We were unable to exclude observations from our analysis that were missed for valid clinical or logistic reasons, such as when patients were away from the ward (e.g. for radiological or surgical procedures). A previous study in the same hospital also showed that nursing staff are more reluctant to wake patients at night [33], which could account for some missed observations.

Although we adjusted for daily staffing requirements by incorporating admission rates and the proportion of higher acuity patients into our multi-level models, we were unable to account for other demands on nursing staffing (e.g. personal care needs). However, this potential source of bias would tend to underestimate the effect of low staffing, if staffing is increased when demand is high.

For peer review only - http://bmjopen.bmj.com/site/about/guidelines.xhtml

CONCLUSIONS

This is the first study to demonstrate an association between nurse staffing levels and an objective measure of complete and timely care in relation to monitoring patients' vital signs, a key mechanism hypothesised to explain the link between low nurse staffing and increased mortality. Compliance with vital signs monitoring schedules is lower when levels of registered nurse and nursing assistant staff are lower, although substantial increases in numbers of staff would be required to effect meaningful increase in adherence. It is likely that other factors, such as clinical judgement, are the main drivers of non-adherence.

FUNDING

This project was funded by the NIHR Health Services and Delivery Research Programme (HS&DR 13/114/17). This paper draws on research and data reported in more detail in the NIHR Journal's Library Publication:

P. Griffiths J. Ball, K. Bloor, D. Böhning, J. Briggs, C. Dall'Ora, A. De Iongh, J. Jones, C. Kovacs, A. Maruotti, P. Meredith, D. Prytherch, A. R. Saucedo, O. Redfern, P. Schmidt, N. Sinden and G. Smith. "Nurse staffing levels, missed vital signs and mortality in hospitals: retrospective longitudinal observational study." Health Services and Delivery Research Journal 2018; 6(38).

DATA AVAILABILITY

Due to conditions attached to data sharing agreements between the hospital Trust and the research team, we are unable to share original data. Any queries should be submitted to the corresponding author in the first instance.

DISCLAIMER

The views expressed are those of the authors and not necessarily those of the NHS, the NIHR or the Department of Health and Social Care.

CONTRIBUTIONS OF AUTHORS

PG contributed to the design of the study and acquisition of research funding. PG and OR interpreted the data, and drafted and revised the paper. AM contributed to the design of the study, statistical analysis plan, acquisition of funding and interpretation of data; advised on statistical analysis; contributed to drafting the paper and approved the final manuscript. A R-S and GS contributed to the interpretation of the results and drafting the paper.

For peer review only - http://bmjopen.bmj.com/site/about/guidelines.xhtml

Other members of the Missed Care Study Group contributed to the acquisition of funding and/or data, analysis, interpretation of analysis and approval of the paper.

CONFLICT OF INTERESTS STATEMENT

PM, NS and PES are employees of Portsmouth Hospitals NHS Trust (PHT), which had a royalty agreement with The Learning Clinic (TLC) to pay for the use of PHT intellectual property within the Vitalpac product, which expired during the course of this study. DP and GBS are former employees of PHT. PES, and the wives of DP and GBS, held shares in TLC until 2015. JB's research has previously received funding from TLC through a Knowledge Transfer Partnership. PG was an unpaid member of the advisory group for NHS Improvement's work developing improvement resources for safe staffing in adult inpatient wards.

TABLES

Table 1 Admission characteristics for study participants

**Admissions, N**	138,133
***Emergency admissions,* N (%)**	108,865 (79)
***Elective,* N (%)**	29 268 (21)
***Age* median, (range)**	66.6 (16.0-106)
***Charlson Co-morbidity Index (CCI),* median (range)**	3 (0-98)
***First NEWS,* median (range)**	1 (0-19)
Low (NEWS <3) N (%)	102,674 (74)
Medium (NEWS 3-5) N (%)	27,409 (20)
High (NEWS >5) N (%)	8,050 (6)
***Length of stay* in days, median (range)**	2.73 (0.150-933)
***In-hospital mortality* N (%)**	5,662 (4.1)

For peer review only - http://bmjopen.bmj.com/site/about/guidelines.xhtml

Table 2 Percentage of missed and delayed observations for each of the 32 study wards

Ward	ALL OBSERVATIONS		HIGH ACUITY OBSERVATIONS	
	% Delayed	% Missed	% Delayed	% Missed
SURGICAL: GYNAECOLOGICAL	19.8%	10.4%	38.2%	30.3%
MEDICAL: GASTROENTEROLOGY	42.9%	25.8%	61.0%	52.3%
MEDICAL: CARDIOLOGY/GASTROENTEROLOGY	45.5%	24.7%	62.8%	52.2%
MEDICAL/SURGICAL: CARDIAC HIGH CARE	25.6%	14.0%	48.8%	39.7%
SURGICAL: EMERGENCY ORTHOPAEDIC (SPINAL)	33.2%	19.1%	46.9%	37.4%
MEDICAL: GENERAL	40.9%	22.4%	64.1%	55.1%
MEDICAL: GENERAL	30.8%	14.4%	39.8%	32.0%
SURGICAL: EMERGENCY ORTHOPAEDIC (HEAD INJURY)	24.3%	10.9%	44.4%	35.9%
SURGICAL: ELECTIVE ORTHOPAEDIC	21.3%	11.9%	29.8%	23.3%
SURGICAL: OLDER PEOPLE	31.9%	17.4%	39.7%	30.7%
SURGICAL: GENERAL UROLOGY, VASCULAR, PLASTIC	29.2%	15.0%	45.9%	34.4%
SURGICAL: HEAD & NECK	29.1%	12.9%	53.2%	43.7%
SURGICAL: GENERAL, UPPER GI	21.0%	8.8%	36.3%	27.9%
SURGICAL: GENERAL/COLORECTAL	23.5%	10.6%	44.1%	36.2%
MEDICAL: RESPIRATORY HIGH CARE AND STEP DOWN	52.6%	38.5%	71.8%	64.2%
MEDICAL: RESPIRATORY	47.7%	30.7%	63.5%	53.4%
REHABILITATION: NEURO	61.2%	45.2%	56.2%	47.9%
MEDICAL : OLDER PEOPLE	28.0%	15.2%	51.0%	41.8%
REHABILITATION: STROKE (OLDER PEOPLE)	52.5%	35.8%	53.1%	44.1%
MEDICAL: ACUTE STROKE	40.9%	19.3%	58.7%	49.9%
MEDICAL: RADIOTHERAPY HAEMATOLOGY / ONCOLOGY	24.3%	11.5%	54.8%	44.7%
MEDICAL : OLDER PEOPLE	32.8%	16.4%	56.2%	45.5%
MEDICAL : OLDER PEOPLE	39.6%	19.0%	58.4%	47.3%
MEDICAL : OLDER PEOPLE	38.2%	20.6%	59.7%	49.7%
MEDICAL : OLDER PEOPLE	36.7%	17.2%	60.6%	48.9%
MEDICAL/SURGICAL: ELECTIVE & INVESTIGATIONS	18.7%	8.8%	37.1%	30.5%
MEDICAL: RENAL HIGH CARE	25.6%	13.2%	45.8%	36.4%
MEDICAL: RENAL	21.9%	10.8%	46.5%	38.4%
SURGICAL: RENAL TRANSPLANT	16.3%	7.6%	38.3%	31.5%
MEDICAL: EMERGENCY ADMISSIONS	19.7%	9.1%	50.4%	39.0%
SURGICAL: ADMISSIONS	15.4%	5.6%	39.1%	31.7%
SURGICAL: HIGH CARE	9.8%	5.5%	31.0%	22.4%

For peer review only - http://bmjopen.bmj.com/site/about/guidelines.xhtml

Table 3 Mixed-effects Poisson regression: Association between staffing and all missed observations with (A) and without (B) inclusion of a linear interaction term between RN and NA staffing levels. IRR = incidence rate ratio Model A: AIC 215974 BIC 216033 Model B: AIC 216062 BIC 216112

MODEL	A			B		
	IRR	95% confidence interval	p-value	IRR	95% confidence interval	p-value
**RN staffing**	0.983	0.979-0.987	< 0.001	0.981	0.977-0.985	< 0.001
**NA staffing**	0.954	0.949-0.958	< 0.001	0.957	0.952-0.961	< 0.001
**Patient turnover**	1.01	1.01-1.01	< 0.001	1.01	1.01-1.02	< 0.001
**Observations in higher acuity patients**	4.83	4.68-4.99	< 0.001	4.8	4.65-4.96	< 0.001
**RN staffing x NA staffing**				1.01	1.01-1.01	< 0.001

Table 4 Mixed-effects Poisson regression: Association between staffing and high acuity missed observations with (a) and without (b) inclusion of a linear interaction term between RN and NA staffing levels. IRR = incidence rate ratio. Model A: AIC 76747; BIC; 76796 Model B: AIC 76749; BIC 76806

MODEL	A			B		
	IRR	95% confidence interval	p-value	IRR	95% confidence interval	p-value
**RN Staffing**	0.982	0.972-0.992	< 0.001	0.982	0.972-0.992	< 0.001
**NA Staffing**	1	0.990-1.01	0.822	1	0.991-1.01	0.791
**Patient Turnover**	0.997	0.988-1.01	0.59	0.997	0.988-1.01	0.582
**Observations in higher acuity patients**	1.01	0.936-1.09	0.769	1.01	0.937-1.09	0.747
**RN staffing x NA staffing**				0.999	0.994-1.00	0.64

For peer review only

FIGURES

Figure 1 Partial dependency plots showing interaction effects between levels of registered nurse and nursing assistant (NA) for all missed observations

For peer review only

For peer review only - http://bmjopen.bmj.com/site/about/guidelines.xhtml

REFERENCES

1 Keogh B. Review into the quality of care and treatment provided by 14 hospital trusts in England: overview report. *NHS* 2013.

2 Page A. *Keeping patients safe: Transforming the work environment of nurses*. National Academies Press 2004.

3 Clarke SP. Failure to rescue: lessons from missed opportunities in care. *Nurs Inq* 2004;**11**:67–71. doi:10.1111/j.1440-1800.2004.00210.x

4 Griffiths P, Recio-Saucedo A, Dall'Ora C, *et al.* The association between nurse staffing and omissions in nursing care: A systematic review. *J Adv Nurs* 2018;**74**:1474–87. doi:10.1111/jan.13564

5 Griffiths P, Ball J, Drennan J, *et al.* Nurse staffing and patient outcomes: Strengths and limitations of the evidence to inform policy and practice. A review and discussion paper based on evidence reviewed for the National Institute for Health and Care Excellence Safe Staffing guideline develo. *Int J Nurs Stud* 2016;**63**:213–25. doi:10.1016/j.ijnurstu.2016.03.012

6 Kalisch BJ, Landstrom GL, Hinshaw AS. Missed nursing care: a concept analysis. *J Adv Nurs* 2009;**65**:1509–17. doi:10.1111/j.1365-2648.2009.05027.x

7 Schubert M, Glass TR, Clarke SP, *et al.* Validation of the Basel Extent of Rationing of Nursing Care Instrument. *Nurs Res* 2007;**56**:416–24. doi:10.1097/01.NNR.0000299853.52429.62

8 Lucero RJ, Lake ET, Aiken LH. Nursing care quality and adverse events in US hospitals. *J Clin Nurs* 2010;**19**:2185–95. doi:10.1111/j.1365-2702.2010.03250.x

9 Ausserhofer D, Zander B, Busse R, *et al.* Prevalence, patterns and predictors of nursing care left undone in European hospitals: results from the multicountry cross-sectional RN4CAST study. *BMJ Qual Saf* 2014;**23**:126–35. doi:10.1136/bmjqs-2013-002318

10 Ball JE, Bruyneel L, Aiken LH, *et al.* Post-operative mortality, missed care and nurse staffing in nine countries: A cross-sectional study. *Int J Nurs Stud* 2018;**78**:10–5. doi:10.1016/j.ijnurstu.2017.08.004

11 National Institute for Health and Care Excellence. Acutely ill patients in hospital: Recognition of and response to acute illness in adults in hospital. NICE guideline (50). London: 2007.

For peer review only - http://bmjopen.bmj.com/site/about/guidelines.xhtml

12 Royal College of Physicians (London). National Early Warning Score (NEWS): Standardising the assessment of acute illness severity in the NHS. Report of a working party. 2012.

13 National Patient Safety Agency. Recognising and responding appropriately to early signs of deterioration in hospitalised patients. 2007. doi:accessed 03.04.14

14 Smith GB, Prytherch DR, Schmidt PE, *et al.* A review, and performance evaluation, of single-parameter 'track and trigger' systems. *Resuscitation* 2008;**79**:11–21. doi:10.1016/j.resuscitation.2008.05.004

15 Hands C, Reid E, Meredith P, *et al.* Patterns in the recording of vital signs and early warning scores: compliance with a clinical escalation protocol. *BMJ Qual Saf* 2013;**22**:719–26. doi:10.1136/bmjqs-2013-001954

16 Recio-Saucedo A, Maruotti A, Griffiths P, *et al.* Relationships between healthcare staff characteristics and the conduct of vital signs observations at night: Results of a survey and factor analysis. *Nurs Open* 2018;**5**:621–33. doi:10.1002/nop2.179

17 Antonakis J, Bendahan S, Jacquart P, *et al.* On making causal claims: A review and recommendations. *Leadersh Q* 2010;**21**:1086–120. doi:10.1016/j.leaqua.2010.10.010

18 Smith GB, Prytherch DR, Schmidt P, *et al.* Hospital-wide physiological surveillance-A new approach to the early identification and management of the sick patient. *Resuscitation* 2006;**71**:19–28. doi:10.1016/j.resuscitation.2006.03.008

19 R Core Team. R: A Language and Environment for Statistical Computing. 2017.

20 Bates D, Mächler M, Bolker B, *et al.* Fitting Linear Mixed-Effects Models Using lme4. *J Stat Softw* 2015;**67**. doi:10.18637/jss.v067.i01

21 Smith GB. In-hospital cardiac arrest: Is it time for an in-hospital 'chain of prevention'? *Resuscitation* 2010;**81**:1209–11. doi:10.1016/j.resuscitation.2010.04.017

22 Griffiths P, Jones S, Bottle A. Is "failure to rescue" derived from administrative data in England a nurse sensitive patient safety indicator for surgical care? Observational study. *Int J Nurs Stud* 2013;**50**:292–300. doi:10.1016/j.ijnurstu.2012.10.016

23 Zander B, Dobler L, Bäumler M, *et al.* Implizite Rationierung von Pflegeleistungen in deutschen Akutkrankenhäusern – Ergebnisse der internationalen Pflegestudie RN4Cast. *Das Gesundheitswes* 2014;**76**:727–34. doi:10.1055/s-0033-1364016

24 JAMES J, BUTLER-WILLIAMS C, HUNT J, *et al.* Vital signs for vital people: an exploratory study into the role of the Healthcare Assistant in recognising, recording and

For peer review only

responding to the acutely ill patient in the general ward setting. *J Nurs Manag* 2010;**18**:548–55. doi:10.1111/j.1365-2834.2010.01086.x

25 Griffiths P, Saucedo AR, Schmidt P, *et al.* Vital signs monitoring in hospitals at night. *Nurs Times* 2015;**111**:16–7.

26 Duffield CM, Twigg DE, Pugh JD, *et al.* The Use of Unregulated Staff: Time for Regulation? *Policy, Polit Nurs Pract* 2014;**15**:42–8. doi:10.1177/1527154414529337

27 Koyner JL, Carey KA, Edelson DP, *et al.* The Development of a Machine Learning Inpatient Acute Kidney Injury Prediction Model*. *Crit Care Med* 2018;**46**:1070–7. doi:10.1097/CCM.0000000000003123

28 Smith GB, Prytherch DR, Meredith P, *et al.* The ability of the National Early Warning Score (NEWS) to discriminate patients at risk of early cardiac arrest, unanticipated intensive care unit admission, and death. *Resuscitation* 2013;**84**:465–70. doi:10.1016/j.resuscitation.2012.12.016

29 Ball JE, Murrells T, Rafferty AM, *et al.* 'Care left undone' during nursing shifts: associations with workload and perceived quality of care. *BMJ Qual Saf* 2014;**23**:116–25. doi:10.1136/bmjqs-2012-001767

30 Smith GB, Recio-Saucedo A, Griffiths P. The measurement frequency and completeness of vital signs in general hospital wards: An evidence free zone? *Int J Nurs Stud* Published Online First: July 2017. doi:10.1016/j.ijnurstu.2017.07.001

31 Mainz J. Developing evidence-based clinical indicators: a state of the art methods primer. *Int J Qual Heal Care* 2003;**15**:5i – 11. doi:10.1093/intqhc/mzg084

32 Mainz J. Defining and classifying clinical indicators for quality improvement. *Int J Qual Heal Care* 2003;**15**:523–30. doi:10.1093/intqhc/mzg081

33 Hope J, Recio-Saucedo A, Fogg C, *et al.* A fundamental conflict of care: Nurses' accounts of balancing patients' sleep with taking vital sign observations at night. *J Clin Nurs* 2018;**27**:1860–71. doi:10.1111/jocn.14234

For peer review only

For peer review only - http://bmjopen.bmj.com/site/about/guidelines.xhtml

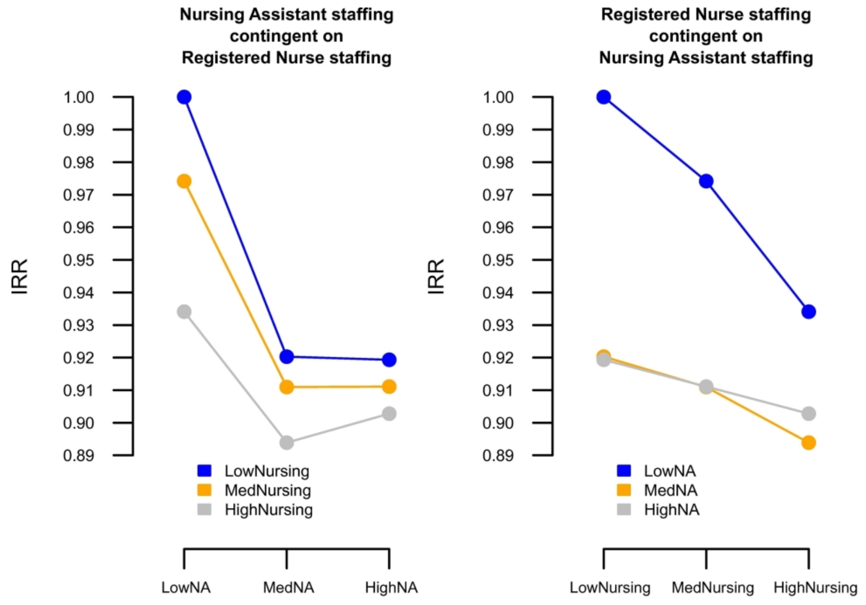

9

Figure 1 Partial dependency plots showing interaction effects between levels of registered nurse and nursing assistant (NA) for all missed observations

191x271mm (300 x 300 DPI)

SUPPLEMENTARY MATERIAL

A1 Summary of study hospital escalation and vital sign monitoring protocol

Early warning Score	Risk category	Max interval between observations	Nurse Actions	Doctor Actions
**0-1**	Low	6 hours /<br>12 Hours If stable for 6 hours	(None specified – observations as per schedule)	
**2**	Low	6 hours	(None specified – observations as per schedule)	
**3-5**	Medium	4 hours	Inform nurse in charge	
**<6, but with one or more individual triggers**	High	4 hours	Registered nurse to inform doctor (FY2 / SHO)	See patient within 2 hours
**6**	High	4 hours	Registered nurse to inform doctor (FY2 / SHO)	See patient within 2 hours
**7-8**	High	1 hour	Registered nurse to inform doctor (FY2 / SHO)<br>Consider continuous monitoring	See patient within 30 minutes<br>Call SpR / outreach (after 8.30 SpR / ICU)
**9+**	Critical	30 minutes	Registered nurse to inform doctor (SpR)<br>Consider continuous monitoring	See patient within 15 minutes<br>Call SpR / outreach (after 8.30 SpR / ICU)

Extreme values on any one parameter may trigger a higher level of escalation than otherwise indicated

Full policy can be found at :
http://www.porthosp.nhs.uk/about-us/policies-and-guidelines/policies/Clinical/Deteriorating%20Patient%20Policy%20-%20Management.doc (accessed 13/1/2018).

Adapted from Griffiths P, Ball J, Bloor K, et al. Nurse staffing levels, missed vital signs and mortality in hospitals: retrospective longitudinal observational study. Heal Serv Deliv Res 2018;6:1–120.

For peer review only

A2 Study flowchart

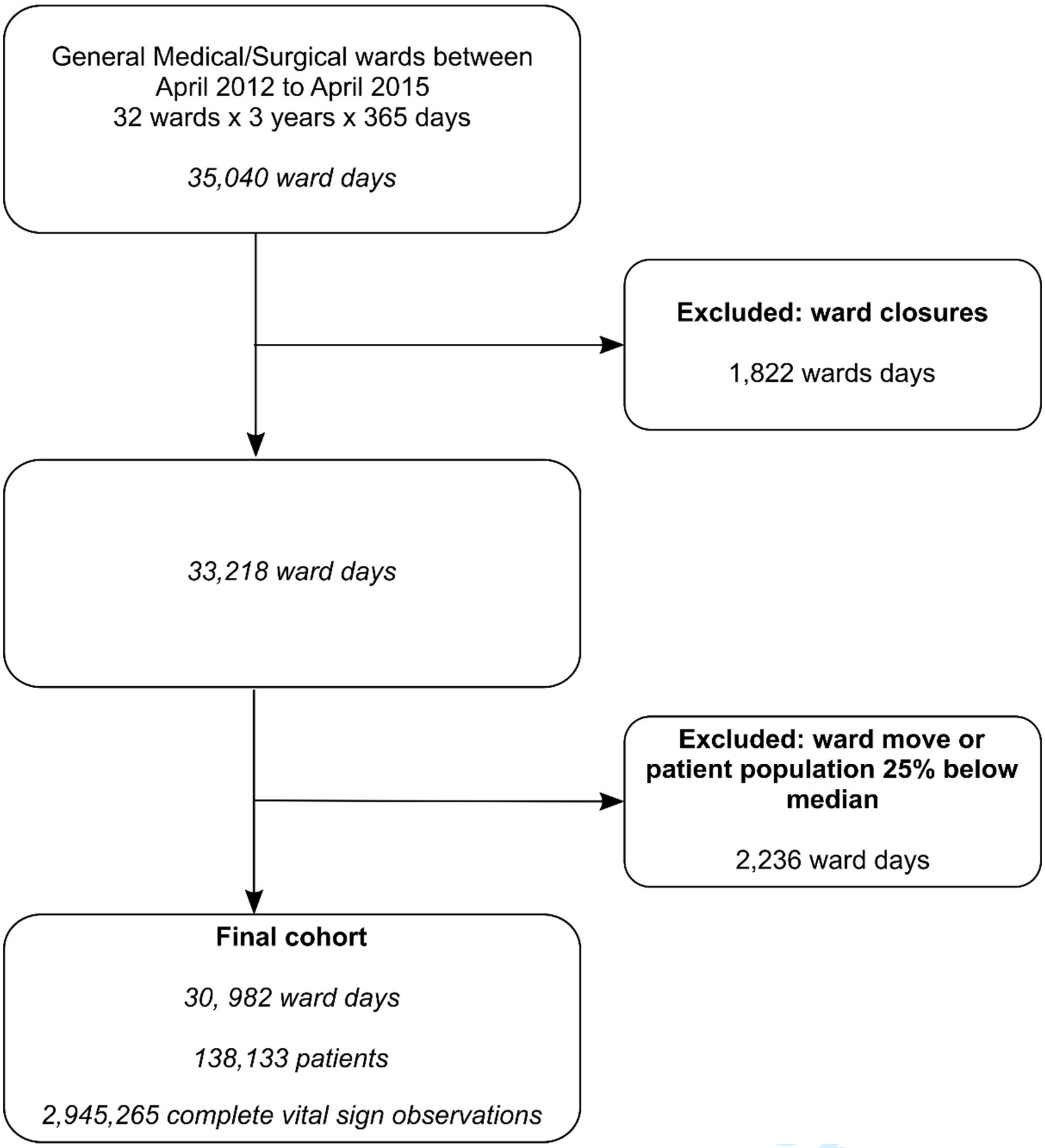

A3 Mean staffing per ward

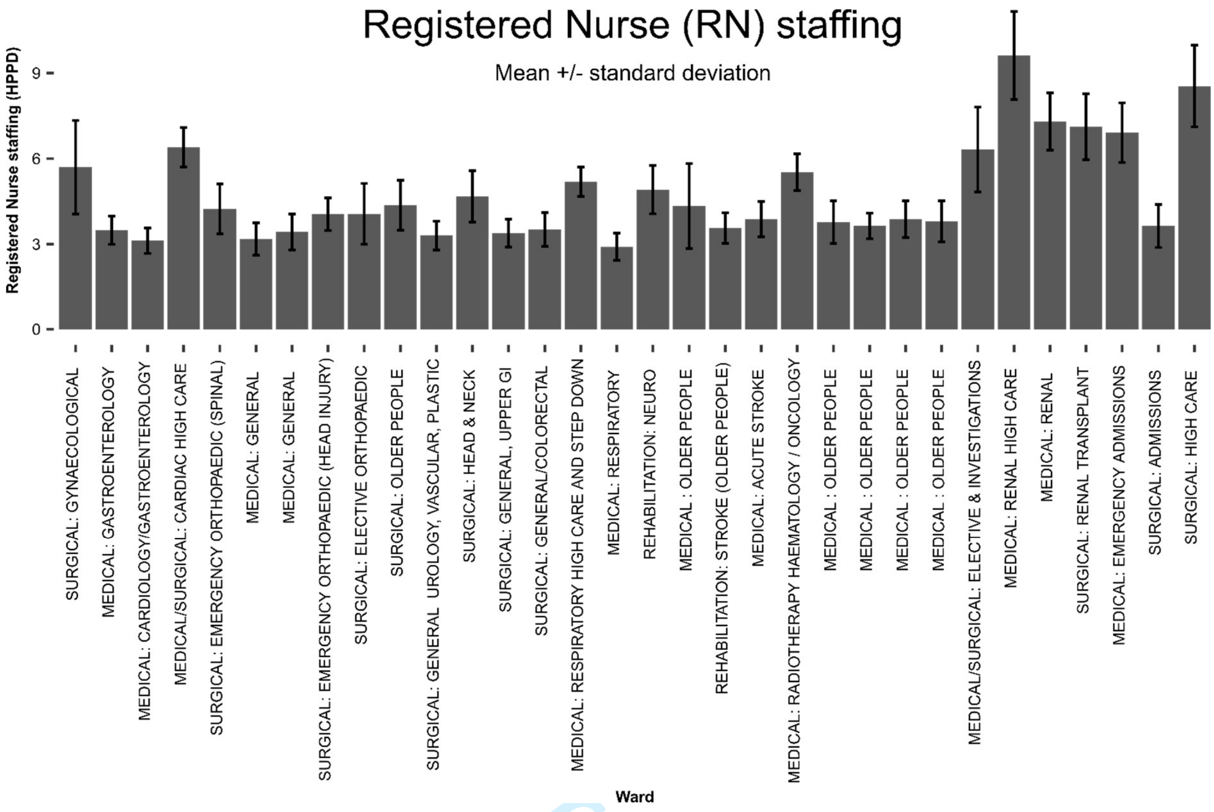

A4 Missed observations (low and medium acuity)

The tables below show the relationship of staffing levels with respect to the primary outcome (missed observations) for low and medium acuity observations.

Table A4a Low acuity

	IRR	95% confidence Interval	p-value
**RN staffing**	0.98	0.973-0.986	< 0.001
**NA staffing**	0.933	0.926-0.939	< 0.001
**Patient turnover**	1.04	1.03-1.04	< 0.001
**Higher acuity patients**	2.03	1.93-2.14	< 0.001
**RN staffing x NA staffing**	1.02	1.02-1.02	< 0.001

review only

Table A4b Medium acuity

	IRR	95% confidence Interval	p-value
**RN staffing**	0.977	0.971-0.984	< 0.001
**NA staffing**	0.964	0.957-0.971	< 0.001
**Patient turnover**	0.989	0.984-0.995	< 0.001
**Higher acuity patients**	0.641	0.609-0.676	< 0.001
**RN staffing x NA staffing**	1.01	1.00-1.01	< 0.001

A5 Delayed observations

The tables below show the relationship of staffing levels with respect to the secondary outcome (delayed observations).

Table A5a All observations

	IRR	95% confidence Interval	p-value
**RN staffing**	0.984	0.981-0.987	< 0.001
**NA staffing**	0.98	0.976-0.983	< 0.001
**Patient turnover**	1	1.00-1.01	0.0186
**Higher acuity patients**	2.23	2.18-2.28	0
**RN staffing x NA staffing**	1.01	1.00-1.01	< 0.001

Table A5b High acuity observations

	IRR	95% confidence Interval	p-value
**RN staffing**	0.987	0.978-0.996	0.0043
**NA staffing**	1	0.993-1.01	0.55
**Patient turnover**	0.996	0.987-1.00	0.38
**Higher acuity patients**	1.03	0.960-1.10	0.415
**RN staffing x NA staffing**	1	0.995-1.00	0.943

A6 Missed observations (tertiles of nurse staffing levels)

The table below shows the model used to explore interactions between NA and RN staffing groups (see Figure 1 in main manuscript).

For peer review only

Table A6 Mixed-effects Poisson regression with staffing variables modelled as tertiles: Association between staffing and all missed observations AIC: 215,999 BIC: 216,099

	IRR	95% confidence interval	p-value
**RN staffing Q2**	0.974	0.963-0.985	p < 0.001
**RN staffing Q3**	0.934	0.923-0.946	p < 0.001
**NA staffing Q2**	0.92	0.910-0.931	p < 0.001
**NA staffing Q3**	0.919	0.908-0.931	p < 0.001
**Patient turnover**	1.01	1.01-1.02	p < 0.001
**Higher acuity patients**	4.83	4.68-4.99	0
**RN staffing Q2 x NA staffing Q2**	1.02	0.999-1.03	0.0603
**RN staffing Q3 x NA staffing Q2**	1.04	1.02-1.06	p < 0.001
**RN staffing Q2 x NA staffing Q3**	1.02	1.00-1.04	0.0525
**RN staffing Q3 x NA staffing Q3**	1.05	1.03-1.07	p < 0.001

For peer review only

2STROBE 2007 (v4) Statement—Checklist of items that should be included in reports of *cohort studies*

Section/Topic	Item #	Recommendation	Reported on page #
**Title and abstract**	1	(*a*) Indicate the study's design with a commonly used term in the title or the abstract	2
		(*b*) Provide in the abstract an informative and balanced summary of what was done and what was found	2
**Introduction**			
Background/rationale	2	Explain the scientific background and rationale for the investigation being reported	4-5
Objectives	3	State specific objectives, including any prespecified hypotheses	5
**Methods**			
Study design	4	Present key elements of study design early in the paper	5
Setting	5	Describe the setting, locations, and relevant dates, including periods of recruitment, exposure, follow-up, and data collection	5
Participants	6	(*a*) Give the eligibility criteria, and the sources and methods of selection of participants. Describe methods of follow-up	5-6
		(*b*) For matched studies, give matching criteria and number of exposed and unexposed	N/A
Variables	7	Clearly define all outcomes, exposures, predictors, potential confounders, and effect modifiers. Give diagnostic criteria, if applicable	6-7
Data sources/ measurement	8*	For each variable of interest, give sources of data and details of methods of assessment (measurement). Describe comparability of assessment methods if there is more than one group	*6-7*
Bias	9	Describe any efforts to address potential sources of bias	5
Study size	10	Explain how the study size was arrived at	6
Quantitative variables	11	Explain how quantitative variables were handled in the analyses. If applicable, describe which groupings were chosen and why	6,7
Statistical methods	12	(*a*) Describe all statistical methods, including those used to control for confounding	7
		(*b*) Describe any methods used to examine subgroups and interactions	7
		(*c*) Explain how missing data were addressed	N/A
		(*d*) If applicable, explain how loss to follow-up was addressed	N/A
		(*e*) Describe any sensitivity analyses	N/A
**Results**			

For peer review only

Participants	13*	(a) Report numbers of individuals at each stage of study—eg numbers potentially eligible, examined for eligibility, confirmed eligible, included in the study, completing follow-up, and analysed	Figure 1
		(b) Give reasons for non-participation at each stage	Figure 1
		(c) Consider use of a flow diagram	Figure 1
Descriptive data	14*	(a) Give characteristics of study participants (eg demographic, clinical, social) and information on exposures and potential confounders	8, Table 1
		(b) Indicate number of participants with missing data for each variable of interest	N/A
		(c) Summarise follow-up time (eg, average and total amount)	Table 1
Outcome data	15*	Report numbers of outcome events or summary measures over time	8, Table 1, Table 2
Main results	16	(*a*) Give unadjusted estimates and, if applicable, confounder-adjusted estimates and their precision (eg, 95% confidence interval). Make clear which confounders were adjusted for and why they were included	Table 3
		(*b*) Report category boundaries when continuous variables were categorized	Figure 2, Supp A2,
		(*c*) If relevant, consider translating estimates of relative risk into absolute risk for a meaningful time period	N/A
Other analyses	17	Report other analyses done—eg analyses of subgroups and interactions, and sensitivity analyses	9, Supp file
**Discussion**			
Key results	18	Summarise key results with reference to study objectives	9
**Limitations**			
Interpretation	20	Give a cautious overall interpretation of results considering objectives, limitations, multiplicity of analyses, results from similar studies, and other relevant evidence	9-11
Generalisability	21	Discuss the generalisability (external validity) of the study results	10
**Other information**			
Funding	22	Give the source of funding and the role of the funders for the present study and, if applicable, for the original study on which the present article is based	11

*Give information separately for cases and controls in case-control studies and, if applicable, for exposed and unexposed groups in cohort and cross-sectional studies.

Note: An Explanation and Elaboration article discusses each checklist item and gives methodological background and published examples of transparent reporting. The STROBE checklist is best used in conjunction with this article (freely available on the Web sites of PLoS Medicine at http://www.plosmedicine.org/, Annals of Internal Medicine at http://www.annals.org/, and Epidemiology at http://www.epidem.com/). Information on the STROBE Initiative is available at www.strobe-statement.org.

For peer review only
